# Hypoxia-induced signaling in the cardiovascular system: pathogenesis and therapeutic targets

**DOI:** 10.1038/s41392-023-01652-9

**Published:** 2023-11-20

**Authors:** Yongchao Zhao, Weidong Xiong, Chaofu Li, Ranzun Zhao, Hao Lu, Shuai Song, You Zhou, Yiqing Hu, Bei Shi, Junbo Ge

**Affiliations:** 1 Department of Cardiology, Affiliated Hospital of Zunyi Medical University, Zunyi, 563000 China; 2 Department of Cardiology, Zhongshan Hospital, Fudan University, Shanghai Institute of Cardiovascular Diseases, Shanghai, 200032 China; 3 Key Laboratory of Viral Heart Diseases, National Health Commission, Shanghai, 200032 China; 4 Key Laboratory of Viral Heart Diseases, Chinese Academy of Medical Sciences, Shanghai, 200032 China; 5 National Clinical Research Center for Interventional Medicine, Shanghai, 200032 China; 6 Shanghai Clinical Research Center for Interventional Medicine, Shanghai, 200032 China; 7 Institutes of Biomedical Sciences, Fudan University, Shanghai, 200032 China

**Keywords:** Molecular biology, Cardiology, Molecular medicine

## Abstract

Hypoxia, characterized by reduced oxygen concentration, is a significant stressor that affects the survival of aerobic species and plays a prominent role in cardiovascular diseases. From the research history and milestone events related to hypoxia in cardiovascular development and diseases, The "hypoxia-inducible factors (HIFs) switch" can be observed from both temporal and spatial perspectives, encompassing the occurrence and progression of hypoxia (gradual decline in oxygen concentration), the acute and chronic manifestations of hypoxia, and the geographical characteristics of hypoxia (natural selection at high altitudes). Furthermore, hypoxia signaling pathways are associated with natural rhythms, such as diurnal and hibernation processes. In addition to innate factors and natural selection, it has been found that epigenetics, as a postnatal factor, profoundly influences the hypoxic response and progression within the cardiovascular system. Within this intricate process, interactions between different tissues and organs within the cardiovascular system and other systems in the context of hypoxia signaling pathways have been established. Thus, it is the time to summarize and to construct a multi-level regulatory framework of hypoxia signaling and mechanisms in cardiovascular diseases for developing more therapeutic targets and make reasonable advancements in clinical research, including FDA-approved drugs and ongoing clinical trials, to guide future clinical practice in the field of hypoxia signaling in cardiovascular diseases.

## Introduction

Cardiovascular diseases contribute significantly to the global burden of disease and are closely linked to hypoxia, or oxygen deprivation. As aerobic organisms emerged and thrived, oxygen played an indelible role in the survival and evolution of these organisms, especially higher life forms. The hypoxia-inducible factors (HIFs), serving as a central molecule in the hypoxia response, exhibits a remarkable degree of conservation across different species. In the human physiological state, the oxygen partial pressure (pO_2_) fluctuates within and across organs due to tissue structure and the presence of arterioles and capillaries. Oxygen levels exhibit variations, ranging from as low as 0.5% in the large intestine to a maximum of 13% in the lungs. Nevertheless, several vital organs maintain tissue oxygen levels within the range of ~3–7%.^1^ In addition, the increased oxygen demand resulting from cellular hypertrophy in a physiological state may not be adequately met by the oxygen tension supplied by blood vessels (or neovascularization), or it could lead to a pseudohypoxic state within cells caused by a hypermetabolic state. These conditions have the potential to trigger subsequent maladaptive pathological processes. The HIFs pathways are crucial in the field of cardiovascular biology.^2,3^ In the developing heart, the occurrence of gestational hypoxia initiates specific pathways controlled by HIF-1, which are crucial for the formation of heart chambers and septum.^4,5^ The adult heart encounters recurring periods of hypoxia, which occur naturally (for example, at high altitudes and during physical activity) as well as in abnormal situations (like ischemia, cardiomyocyte hypertrophy, inflammation, and fibrosis).^6^

Irrespective of the stimuli, the activation of HIFs results in the stabilization of the HIF-α subunit. Normally, this subunit has an exceptionally brief half-life of less than 5 min. Conversely, the β subunit maintains a consistent level of expression under normal oxygen conditions.^7^ Under normal oxygen conditions, HIF prolyl hydroxylases (PHDs) and asparaginyl hydroxylase (Factor Inhibiting HIF or FIH) modify HIF-1α, leading to its degradation and suppressing transcriptional activity. In hypoxic conditions, these modifications are constrained, enabling HIF-1α to translocate to the nucleus, form a dimer with HIF-1β, and bind to hypoxia response elements (HREs) to promote transcription.^8,9^ Another isoform of HIF, known as HIF-2α, detects oxygen levels and assumes a narrower yet crucial function, particularly within the vasculature.^10^ The regulation of HIFs differs significantly, with HIF-2α generally exhibiting stabilization at relatively higher levels of oxygen compared to HIF-1α.^11^ Despite their similarities, they also exert distinct regulation on diverse target genes when interacting with alternative transcription factors and coregulators.^12,13^ Generally, HIF-1α activates glycolytic genes, reduces oxygen use, and lowers reactive oxygen species (ROS) production, while HIF-2α enhances erythropoietin (EPO) synthesis, and iron metabolism, and regulates fatty acid synthesis and uptake, inflammation, fibrosis, and vascular tumors.^14,15^

HIFs are central molecules in the hypoxic response.^1^ Due to the intricate interplay between oxygen and internal/external environmental factors, the roles played by different HIF isoforms can vary with spatial and temporal specificity. This feature may stem from inherent factors such as natural selection in high-altitude geographical environments or modifications from acquired factors like epigenetics. It may also be associated with the organism’s acute or chronic response to hypoxic conditions over a certain period of time. Furthermore, the interaction between oxygen and circadian rhythms caused by the Earth’s rotation and hibernation rhythms induced by seasonal changes contribute to the rise and fall of civilizations. Cardiovascular diseases continue to be the primary causes of death globally, significantly impacting overall health and leading to excessive healthcare expenses.^16^ Additionally, these diseases exhibit characteristics of both acute events, such as heart attacks, and chronic conditions like chronic coronary total occlusion, and heart failure. Furthermore, with the emergence of the holistic concept of panvascular medicine,^17,18^ it has become increasingly crucial to explore the interplay between HIFs and the environment, particularly in relation to the commonalities and distinguishing features of cardiovascular diseases.

This review retrospectively summarized the research history and milestones of hypoxia in cardiovascular development and diseases. Then, the "HIFs switch" was discussed in terms of temporal and spatial dimensions, including the occurrence and progression of hypoxia (gradual decrease of oxygen concentration), the manifestations of acute and chronic hypoxia, and the geographical features of hypoxia (natural selection at high altitude). Also, the impact of epigenetics on cardiovascular hypoxia signaling pathways was highlighted. Furthermore, we explored the role of hypoxia signaling pathways in natural rhythms and their associations with different tissues and organs within the cardiovascular system, as well as their interactions with other systems. This provides a foundation for understanding the multi-level regulatory signaling pathways and mechanisms of hypoxia signaling in cardiovascular diseases. Finally, we summarize therapeutic targets and advancements in clinical research for hypoxia signaling in cardiovascular diseases to guide future clinical practice.

## Research history and milestone events of hypoxia in cardiovascular development and diseases

**Fig. 1:**
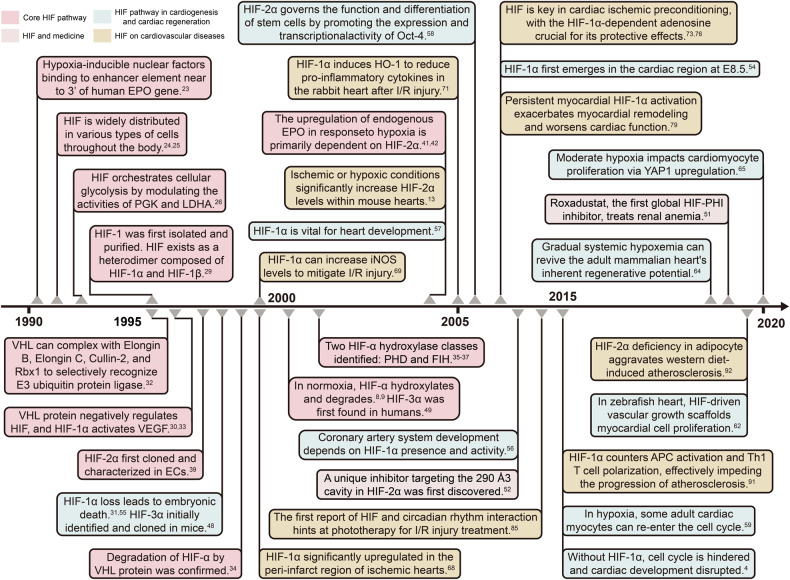
Timeline of key milestones in the development of hypoxia signaling in cardiovascular diseases. Abbreviations: APCs antigen-presenting cells, E8.5 embryonic day 8.5, EPO erythropoietin, FIH factor-inhibiting HIF, HIF hypoxia inducible factor, HO-1 heme oxygenase-1, I/R ischemia/reperfusion, iNOS inducible nitric oxide synthase, IPC ischemic preconditioning, LDHA lactate dehydrogenase, Oct-4 octamer-binding transcription factor 4, PGK phosphoglycerate kinase, PHD prolyl hydroxylases, Rbx1 ring-box protein 1, SIRT Sirtuin, VEGF vascular endothelial growth factor, VHL von Hippel-Lindau, YAP1 Yes-associated protein 1

In 2019, the Nobel Prize in Physiology or Medicine was awarded to American scientist William G. Kaelin Jr., British scientist Sir Peter J. Ratcliffe, and American scientist Gregg L. Semenza, in recognition of their discoveries of how animal cells sense and adapt to changes in oxygen availability. Their research clarified key mechanisms of oxygen adaptation in living organisms, establishing a foundation for comprehending oxygen’s impact on cellular metabolism and physiology. Furthermore, their discoveries offer valuable insights and novel therapeutic avenues for addressing various diseases like anemia, cancer, and cardiovascular issues. The rapid response and adaptation of cells to oxygen changes are vital in most animal cells. Throughout evolution, as animal cells began to form multicellular three-dimensional structures, they needed to autonomously adapt to varying oxygen levels in various ways. Since the 1950s, researchers have recognized that the number of red blood cells (RBCs) vary with cellular oxygen levels. Later findings unveiled the adjustment of EPO and vascular endothelial growth factor (VEGF) to oxygen levels, inaugurating a novel area of research into cellular sensing and response to both regular and hypoxic conditions (Fig. 1).^19^

### The past and present of oxygen sensing

Oxygen sensing is the vital capability of cells and tissues to detect and respond to shifts in intracellular oxygen levels, playing a crucial role in overall physiological processes within an organism, which includes embryonic growth and development, and muscle activity. The changes in oxygen levels in the body can be systemic (e.g., in high-altitude environments) or local (e.g., in localized injuries), both of which can trigger cellular adaptive processes known as hypoxia responses. But how does our body sense oxygen levels, maintain oxygen homeostasis, and achieve balance? To understand this, let’s commence by examining EPO, a cytokine regulating RBCs’ production and influencing blood oxygen-carrying capacity.^20^ Upon perceiving low blood oxygen (anemia) or decreased environmental oxygen (hypoxia), peritubular interstitial cells in kidney, hepatocytes and Ito cells in liver release EPO. This prompts bone marrow hematopoietic stem cells to create more RBCs, enhancing oxygen transport to counter the hypoxic state.^21^ Furthermore, in 1991, Semenza and colleagues identified a conserved DNA sequence related to hypoxia induction at the 3′ end of the *EPO* gene. Connecting this sequence to non-hypoxia-induced genes resulted in their regulation by hypoxia. This sequence was subsequently named the HRE.^22^ It was hypothesized that some factors may be capable of binding to the HREs and regulating the expressions of hypoxia-related genes. The corresponding protein was then detected in the nuclear extract of cells under hypoxia, remained stable only in low oxygen, and was named HIF-1.^23^ In 1993, Semenza et al. further discovered that the stable HIF-1 complex functions not only in EPO-producing cells but also in non-EPO producing cells like those from the skin, lungs, ovaries, and blood vessels, all responsive to hypoxia.^24,25^ This indicates that the oxygen-sensing mechanism is not limited to the *EPO* gene-expressing cells of the kidneys and liver but is likely a phenomenon universally present in the organism. Therefore, HIF-1 emerges as the key to unraveling the interplay between cells and oxygen. Furthermore, in a hypoxia model developed by Ratcliffe et al. in 1994, it was found that HIF-1 directly regulates vital glycolysis enzymes, such as phosphoglycerate kinase and lactate dehydrogenase (LDHA). This marks the era of a metabolic perspective in the comprehension of HIF-1’s regulation of the hypoxic response.^26^ The interconnection between the HIF-related hypoxic signaling pathway and glycolysis metabolism was later regarded as fundamental in establishing the pivotal role of HIFs in both cardiac and tumor metabolic pathways.^6,27^ The spatial structure of the HIF-1 protein was extensively investigated, leading to the first isolation, purification, and characterization of HIF-1 in early 1995. This revealed a heterodimeric complex, comprising a 120 kDa HIF-1α subunit and a 91–94 kDa HIF-1β subunit (also known as aryl hydrocarbon receptor nuclear translocator, ARNT).^28^ Later in the same year, the protein domains of HIF-1α and HIF-1β were also determined.^29^ In 1996, Semenza et al. made another significant discovery by demonstrating that HIF-1α is capable of inducing VEGF, which plays a crucial role in angiogenesis.^30^ Coronary artery disease can lead to myocardial ischemia, resulting in inadequate perfusion and localized hypoxia. VEGF plays a pivotal role in angiogenesis by promoting the development of compensatory processes like collateral vessels or neovascularization, seen in conditions like chronic myocardial ischemia, retinal ischemia, and tumor advancement. Unlike the limited expression of HIF-1-induced EPO in specific cells, various cell types, both primary and cultured, show increased HIF-1-induced VEGF expression in response to hypoxia, providing additional evidence for the widespread and conserved nature of HIF-1. In 1998, in vivo biological function of HIF-1α was confirmed that in the absence of functional HIF-1α, the expression of genes related to vascular development and oxygen dependence was severely impaired, resulting in embryonic lethality.^31^ Collectively, HIF-1α plays a crucial role in an oxygen-sensing mechanism that regulates vascular development and RBC production for oxygen transport in the bloodstream.

**Fig. 2:**
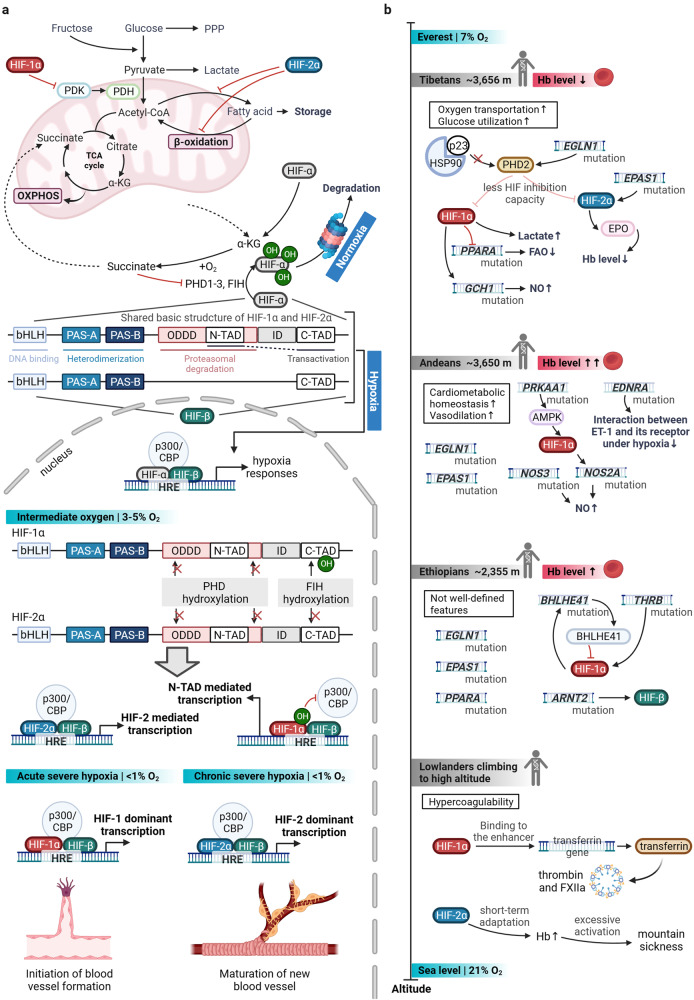
HIFs switch in broader sense. **a** HIFs switch in classical sense. **b** HIFs switch in terms of natural selection. (Created with *BioRender.com*). Abbreviations: acetyl-CoA acetyl coenzyme A, AMPK AMP-activated protein kinase, ARNT2 aryl hydrocarbon receptor nuclear translocator 2, bHLH basic-helix-loop-helix, BHLHE41 basic helix-loop-helix family member e41, C-TAD C-terminal transactivation domain, EDNRA endothelin receptor type A, EGLN1 egl-9 family hypoxia inducible factor 1, EPAS1 endothelial PAS domain protein 1, ET-1 Endothelin-1, FAO fatty acid oxidation, GCH1 GTP cyclohydrolase 1, Hb hemoglobin, HRE hypoxia response element, Hsp90 heat shock protein 90, ID Inhibitory Domain, NO nitric oxide, NOS2A nitric oxide synthase 2A, NOS3 nitric oxide synthase 3, N-TAD N-terminal transactivation domain, ODDD oxygen-dependent degradation domain, OXPHOS oxidative phosphorylation, p300/CBP E1A-binding protein p300/CREB-binding protein, PAS-A Per-Arnt-Sim A, PAS-B Per-Arnt-Sim B, PDH pyruvate dehydrogenase, PDK pyruvate dehydrogenase kinase, PPARA peroxisome proliferator activated receptor alpha, PPP pentose phosphate pathway, PRKAA1 protein kinase AMP-activated catalytic subunit alpha 1, TCA tricarboxylic acid cycle, THRB thyroid hormone receptor beta, α-KG alpha-Ketoglutarate

Research has revealed a phenomenon that the protein level of HIF-1α is strictly regulated by oxygen, being present in hypoxic environments and degraded under normoxic conditions. But what is the underlying mechanism through which the HIF-1α molecule sense oxygen? The answer arose unexpectedly. Around the time Semenza and Ratcliffe groups were exploring the *EPO* gene, Kaelin et al. were delving into von Hippel-Lindau (VHL) syndrome. VHL tumors typically exhibit abnormal neovascularization, coupled with elevated levels of VEGF and EPO, implying a hypoxia pathway involvement in this disease. In 1995, Kaelin et al.’s research^32^ unveiled VHL’s ability to create a complex with Elongin B, Elongin C, Cullin-2, and Rbx1, enabling specific recognition of ubiquitin ligase E3, indicating a crucial link between the VHL complex and the ubiquitin-proteasome system. The following year, Kaelin et al.^33^ found that *VHL* gene mutations in tumor cells led to excessive expression of hypoxia-inducible genes like *VEGF*, which could be reversed by introducing the normal *VHL* gene. Further validation of the relationship between VHL and HIF comes from Ratcliffe et al. in 1999. In normal oxygen levels, VHL binds to HIF-1, marking HIF-1α for quick degradation through ubiquitination. In low oxygen conditions, VHL and HIF-1α separate, stopping the degradation process. In tumor cells with *VHL* gene mutations or deletions, the stability of the HIF-1α protein greatly increases, underscoring VHL’s vital role in HIF-1α degradation.^34^ Following that, a new inquiry emerges: What are the oxygen-dependent mechanism and corresponding structure variations that contribute to the degradation of HIF-1? In 2001, Kaelin and Ratcliffe teams published their groundbreaking research in *Science* magazine in a “back-to-back” format. Both studies revealed that in the presence of oxygen, hydroxylation of proline residues occurs on the HIF-1α peptide, with VHL specifically recognizing and binding to hydroxylated HIF-1α to induce degradation. However, under hypoxic conditions, HIF-1α does not bind to VHL but instead translocates to the cell nucleus, regulating the transcription of various genes for hypoxia adaptation.^8,9^ But what factor governs the oxygen-dependent hydroxylation reaction of HIF-1α? In the subsequent year, Ratcliffe, McKnight, and Kaelin teams independently reported the enzymes responsible for catalyzing the hydroxylation of HIF-1α. These enzymes were identified as prolyl hydroxylases (PHD) and factor-inhibiting HIF (FIH), respectively.^35–37^ In 2002, further research found that the hydroxylation of HIF-1α’s aspartic acid by FIH doesn’t impact its stability. Instead, it hinders the recruitment of coactivators (p300/CBP), leading to reduced transcriptional activation.^38^ In brief, under normoxic conditions, the dual hydroxylation modifications of HIF-1α serve to concurrently suppress protein abundance and transcriptional activity, thus ensuring the strict expression of hypoxia-inducible genes exclusively within hypoxic environments (Fig. 2a).

As HIF exploration deepens, another two new members of the protein family have been discovered. In 1997, the Russell team first cloned and characterized a novel PAS domain transcription factor called endothelial PAS-1 (EPAS1 or HIF-2α) in endothelial cells (ECs). This protein shares a remarkable 48% sequence similarity with HIF-1α and exhibits robust hypoxia-induced expression at the protein level but not at the mRNA level.^39^ In the same year, In that year, the Ema team used a yeast two-hybrid system to isolate a novel cDNA clone from a murine hypothalamus cDNA library. They used the basic Helix-Loop-Helix-PER-ARNT-SIM (bHLH-PAS) domain of ARNT as bait. They discovered that its structure exhibited astonishing similarity to HIF-1α, leading to its designation as HLF (HIF-1α-like factor), which is also synonymous with HIF-2α.^40^ Furthermore, it was discovered that HLF exhibits the highest expression level in the lungs and is closely associated with the expression of VEGF, implying a potential involvement of HLF in lung and vascular development.^40^ Interestingly, initially, it was found that EPO is primarily regulated by HIF-1α under hypoxic conditions. However, mounting evidence suggests that the hypoxic induction of endogenous EPO largely relies on HIF-2α.^41–46^ Histological studies have revealed that the localization of peritubular interstitial cells (such as fibroblasts and ECs) responsible for EPO production coincides with the expression of HIF-2α in renal cells, indirectly suggesting the HIF-2α dependency of EPO expression.^13,47^ In 1998, HIF-3α, the third subtype of HIF-α, was initially identified and cloned in mice,^48^ and later found in humans in 2001.^49^ HIF-3α family, structurally distinct from HIF-1α and HIF-2α, typically generates a polypeptide in certain splicing patterns that counteracts gene expression controlled by HREs.^50^ In 2018, China achieved a global milestone by granting market approval for the oral medication Roxadustat (code-named FG-4592) capsules. This marked the world’s first small-molecule drug within the category of prolyl hydroxylase inhibitors (HIF-PHI) for the treatment of renal anemia.^51^ Roxadustat achieves its objective of correcting anemia by inhibiting PHI enzyme activity via the emulation of one of the substrates of HIF-PHI, ketoglutarate. This action disrupts the balance in the generation and degradation rates of HIF orchestrated by PHI enzymes. The introduction of Roxadustat capsules to the market heralds a new therapeutic avenue for patients afflicted with anemia stemming from chronic kidney disease. Furthermore, given the distinct functions between HIF-1α and HIF-2α, the direct and selective targeting of specific HIF-α subunits with small molecules represents another promising frontier. However, the high similarity between HIF-1α and HIF-2α has hindered the potential for exploring subtype-selective small-molecule inhibitors. In 2009, Gardner et al.^52^ conducted a nuclear magnetic resonance-based ligand-binding assay, screening a pool of 200,000 structurally diverse small molecules. In doing so, they uncovered a unique 290 Å^3^ cavity within the PAS-B domain of HIF-2α capable of accommodating small molecules. This discovery has paved the way for the subsequent development of HIF-2α-specific inhibitors.

### Key events related to HIF pathway in cardiogenesis and cardiac regeneration

The machinery responsible for maintaining the heart’s integral functional form encompasses two crucial modes: the journey from embryonic development to cardiac maturity, and the limited regenerative repair that follows heart injuries. The former involves orchestrating the genesis of heart tissue cells "from scratch," while the latter facilitates the recovery of heart tissue cells "lost and regained" post-damage. Both of these processes are intricately intertwined with intricate responses to oxygen deficiency stress and adaptive mechanisms, in which the spatiotemporal specificity of HIFs is prominently showcased.

#### HIF and heart development

The heart is the first organ to form during mammalian embryogenesis, and its development is crucial for the maturation of the circulatory system and the formation of other organs.^53^ HIF family plays a crucial role in cardiac development. Research indicates that oxygen and the HIF pathway play essential roles in embryogenesis, particularly in the process of cardiac morphogenesis. In mice, cardiac development begins at embryonic day 7.5 (E7.5) with formation of the cardiac crescent and is essentially completed by E15.^10^ At E8.5, HIF-1α first appears in the heart.^54^ By E9.5, HIF-1α is found in the myocardium of the outflow tract as well as the developing ventricles and atria.^4,5^ From E10.5 to E12.5, HIF-1α becomes restricted to the compact myocardium. By E14.5, septation and chamber formation occur, and HIF-1α is no longer detectable in most parts of the heart but remains present in the interventricular septum.^5^ As early as 1998, researchers discovered that the loss of HIF-1α function severely impairs embryonic vascular development, leading to embryonic lethality.^31,55^ Furthermore, the development of the coronary artery vascular system is also dependent on HIF-1α, and its absence may result in clinically significant coronary artery malformations.^56^ A 2003 study also emphasized HIF-1α’s vital role in neural crest migration, ventricular formation, and its significance for cardiac development.^57^ So what is the underlying molecular mechanism of HIF pathways to regulate cardiac development? In 2008, the pivotal role of HIF-1α in regulating the cardiac core transcription factors and myofiber formation was discovered. This occurs through direct activation of core heart transcription factors (Mef2C, and Tbx5), and a connectin critical for sarcomere (titin).^54^ In 2015, researchers found that when HIF-1α is lost, it reduces the expression of MIF and causes DNA damage. These two factors together activate p53 excessively and disrupt the cell cycle, leading to incomplete heart development and developmental halt.^4^ This finding provides an explanation for the observed hypoplastic heart phenotype in HIF-1α-null mice. Another intriguing study revealed that the loss of E3 ubiquitin ligase VHL leads to excessive accumulation of HIF-1α, engendering developmental defects and embryonic lethality. The authors proposed that degradation of HIF-1α in the mid-gestation period within cardiac myocytes promotes a metabolic shift from glycolysis to oxidative metabolism, which is necessary to meet the high-energy demands of a proliferating heart.^5^ Collectively, both deleting or stabilizing HIF-1α in cardiomyocytes can cause underdeveloped hearts, highlighting how HIF-1’s control over gene activity during heart cell growth and differentiation is specific to certain times and locations.

#### HIF and cardiac regeneration

The proliferative capacity of mature mammalian cardiomyocytes is extremely low, and substantial replenishment cannot be achieved in damaged adult hearts. The replenishment of damaged cardiac areas with functional cardiomyocytes is a long-standing goal in regenerative medicine and may benefit from advancements in induced pluripotent stem cell technology. The connection between cellular pluripotency, hypoxia, and HIF activity has been established. Covello et al.^58^ discovered that HIF-2α binds to the promoter of Oct-4, an indispensable transcription factor for maintaining the state of pluripotency in stem cells, inducing the expression and transcriptional activity of Oct-4, thereby regulating stem cell function and/or differentiation. In 2015, Kimura et al.^59^ made an exciting discovery that a small subset of adult cardiomyocytes retains the ability to re-enter the cell cycle under extreme conditions such as hypoxia. Importantly, these hypoxic cardiomyocytes contribute significantly to the formation of new myocardial cells in the adult heart. In contrast to mammals, zebrafish and amphibians efficiently repair damaged hearts through the processes of dedifferentiation and proliferation of existing cardiomyocytes.^60,61^ Ventricular amputation in zebrafish hearts creates a local hypoxic environment at the resection site, which promotes cardiomyocyte dedifferentiation and proliferation. This heart regeneration process is halted when exposed to a hyperoxia environment.^60^ Of note, in the zebrafish heart, HIF-mediated vascular growth acts as a cellular scaffold to facilitate cardiomyocyte regeneration.^62^ The hearts of neonatal mice are capable of regeneration within a few days after birth, and subjecting adult mice to a hypoxic environment also promotes cardiac regeneration.^63,64^ In 2020, Ye et al.^65^ found that moderate hypoxia influences human cardiomyocyte proliferation by upregulating Yes-associated protein 1. These results indicate that hypoxia positively contributes to heart regeneration, which should be taken into account in future strategies for heart regeneration in humans.^66^ Current clinical trials are exploring whether hypoxia can ameliorate cardiac damage in humans. At this time, the mechanisms underlying cardiomyocyte cell cycle reentry under hypoxic conditions are not fully understood, and the role HIFs play in this process remains undefined.

### Key events related to effects of HIF on cardiovascular diseases

Hypoxia is one of the most significant pathogenic factors in cardiovascular diseases. HIF-1α serves as a primary regulator of both physiological and pathological hypoxia and is widely expressed in cardiovascular diseases, heralding the occurrence of various cardiovascular conditions such as atherosclerosis, pulmonary arterial hypertension (PAH), pathological hypertrophy, cardiomyopathy, arrhythmias, congenital heart diseases, heart failure, etc.^6,27^ Myocardial ischemia is one of the most common causes of tissue hypoxia in the heart and can trigger myocardial remodeling. The arterial occlusion caused by atherosclerosis is a significant mechanism leading to ischemia and hypoxia in target organs/tissues. The progression of this occlusion is relatively slow and insidious, with origins traceable back to lipid levels in infancy and early childhood. Furthermore, with the evolution of the concept of panvascular medicine, atherosclerosis is widely acknowledged as the primary shared pathological feature among vascular lesions in panvascular diseases.^17,18^ Panvascular diseases encompass a group of vascular disorders that primarily affect the heart, brain, kidneys, limbs, and major arteries. In a broader sense, they also encompass disorders involving small and microvessels, veins, as well as vascular conditions associated with tumors, diabetes, and immune responses. Furthermore, the lungs, being the organs primarily involved in blood-gas exchange, are the initial sensors of variations in external oxygen levels. Due to their direct exposure to air, they possess a relatively higher oxygen content compared to other organs. Pulmonary hypertension, on another note, can be regarded as a cardiopulmonary coexisting condition, endowing it with unique characteristics in terms of HIFs. Thus, these three types of pathologies will be the focal points of discussion in this section.

#### HIF in myocardial ischemia and heart failure

Ischemic heart disease is caused by coronary artery stenosis or plaque rupture, resulting in reduced myocardial blood flow or oxygen supply.^67^ In 2000, researchers discovered that during the early stages (within the first 24 h) following acute myocardial infarction (MI) or during acute myocardial ischemia, HIF-1α significantly increases in the peri-infarct region of the ischemic hearts, while it remains undetectable in non-ischemic or non-infarcted tissue specimens.^68^ This suggests that HIF-1α may serve as an early molecular marker for myocardial ischemia or infarction. Additionally, studies have revealed that HIF-1α promotes angiogenesis and vascular remodeling by regulating the expression of VEGF, which is critical for the establishment of collateral circulation in myocardial tissue. Similarly, conditions of ischemia or hypoxia lead to a notable elevation in HIF-2α levels within mouse hearts.^13^ HIFs play a vital role in the protection of the heart under ischemic conditions. HIF-1α can increase the levels of inducible nitric oxide synthase (iNOS) to mitigate ischemia-reperfusion (I/R) injury.^69,70^ HIF-1α also induces the production of its downstream target gene, heme oxygenase-1 (HO-1), to mitigate the generation of pro-inflammatory cytokines in the rabbit heart following I/R injury.^71^ Exposing the heart to brief (5 min) ischemia and reperfusion can protect it from subsequent prolonged I/R-induced damage, a phenomenon known as ischemic preconditioning (IPC).^72^ Notably, HIF is a key component of cardiac IPC. Knockdown of HIF-1α eliminates its protective effect in IPC, while knockdown of PHD2 or treatment with the HIF activator dimethyloxalylglycine (DMOG) mimics its protective effect in IPC.^73^ Mechanistically, HIF-1α may activate multiple signaling pathways involved in cardioprotection.^74,75^ Among them, HIF-1α-dependent adenosine signaling pathway is considered as a crucial mechanism underlying the protective effects of HIF-1α-mediated IPC.^73^ Consistent with this hypothesis, adenosine infusion into HIF-1α^+/−^ (partial deficiency of HIF-1α) hearts significantly has prevented I/R injury.^76^ Furthermore, HIF-2α can activate the PI3K/Akt pathway by promoting the transcription of amphiregulin (AREG) and enhancing the expression of epidermal growth factor receptor 1 (ERBB1, AREG is a known ligand for the ERBB1) in cardiomyocytes, providing cardioprotective effects and alleviating I/R injury.^77,78^ Overall, both HIF-1α and HIF-2α enhance the myocardial tissue’s tolerance to ischemic injury.

The mentioned research shows that controlled HIF-1α increase and timely response can safeguard the heart post MI, lessening remodeling and functional decline. However, prolonged HIF-1α activation might worsen remodeling and cardiac function. In 2008, a study using mice with a cardiac-specific *VHL* gene knockout found that sustained expression of HIF-1α initially led to normal growth and cardiac function. However, after five months, these mice exhibited lipid buildup, myocardial fibrosis, remodeling, and heart failure in the cardiomyocytes. This indicated that prolonged HIF-1α presence could negatively impact the heart.^79^ In the same year, excessive HIF-1α specifically in the heart was observed to cause cardiomyopathy and reduce myocardial contractile function.^80^ Further research revealed that chronic inactivation of PHD2 specifically in the heart leads to dilated cardiomyopathy.^81^ In 2012, cardiac-specific overexpression of HIF-1α was confirmed to promote myocardial hypertrophy and worsens cardiac function in a mouse model of pressure overload induced by transverse aortic constriction (TAC).^82^ Collectively, these findings suggest that increased expression of HIF-1α plays a protective role in myocardial ischemia (e.g., acute MI) and I/R, while long-term excessive activation of HIF-1α may cause chronic heart failure.

Furthermore, the severity of myocardial I/R injury may vary throughout the day due to the interaction between circadian rhythms and hypoxic signaling *(which would be further disccussed in the following section)*.^83,84^ Studies have shown that the period circadian regulator 2 (PER2) actively engages in the regulation and maintenance of the biological clock. The absence of PER2 in mice leads to the vanishing of the circadian rhythm associated with HIF-1α. Intriguingly, these *PER2*^*−/−*^ mice exhibit significantly larger areas of MI and can not be protected by IPC, which is an well-known experimental strategy to mitigate myocardial damage regulated by hypoxic signaling.^85^ Therefore, various molecular strategies have been attempted to enhance circadian rhythm signaling to treat I/R injury in animal models, including bright light therapy and blue light therapy.^85–87^ In a mouse model of myocardial I/R injury, bright light therapy was associated with higher levels of PER2 in the heart, increased glycolytic capacity, and a significant reduction in myocardial infarct size.^85^ This study was the first to report the intricate interaction between HIF and circadian rhythm signaling and shed light on potential therapeutic applications of phototherapy in mitigating myocardial I/R injury. Additionally, in mice with EC-specific PER2 deficiency, bright light therapy may maintain EC barrier function by enhancing the transcription of HIF-1α-mediated glycolytic genes through photostimulation.^86^

#### HIF in atherosclerosis

An increasing amount of evidence suggests that hypoxia and oxygen interference are the pathogenesis of atherosclerosis. The hypoxia theory of atherosclerosis posits that an imbalance between oxygen supply and demand in the arterial wall leads to the formation of lesions and plaques.^88^ HIF-1 plays a crucial role in the advancement of atherosclerosis by initiating and promoting foam cell formation, endothelial dysfunction, cellular apoptosis, heightened inflammation, and neovascularization.^89,90^ Within the atherosclerotic lesions in human coronary arteries, hypoxic regions are present, and the progression of the disease is associated with the formation of lipid-laden macrophages (foamy macrophages), local inflammation, and increased vascularization. Immune responses, in particular, drive the pathogenesis of atherosclerosis. It has been found that atherosclerotic lesion formation is associated with an upregulated expression of HIF-1α in atherosclerotic lesions and antigen-presenting cells (APCs) in atherosclerosis-prone mice. By selectively knocking out HIF-1α in CD11c^+^ (as a marker of dendritic cells) APCs, it has been observed that the formation of atherosclerotic plaques and the infiltration of T cells in low-density lipoprotein receptor-deficient (*Ldlr*^*−/−*^) mice are accelerated.^91^ These findings offer unprecedented insights into the function of HIF-1α in APCs in atherosclerosis, and identify HIF-1α to antagonize APC activation and Th1 T cell polarization during atherosclerosis in *Ldlr*^*−/−*^ mice and to attenuate the progression of atherosclerosis. Furthermore, previous research mainly studied HIF-1α’s link to atherosclerosis, while recent studies are exploring HIF-2α’s role. The adipose metabolic dysfunction caused by obesity stands as a primary culprit behind atherosclerosis. HIF-2α in adipocytes has been observed to be upregulated after mild cold exposure at 16 °C and mediate cold-induced thermogenesis. Adipocyte HIF-2α deficiency exacerbated Western-diet-induced atherosclerosis by increasing adipose ceramide levels, which could blunt hepatocyte cholesterol elimination and thermogenesis.^92^ This research highlights adipocyte HIF-2α as a putative pharmacological target for combating atherosclerosis.

Vascular smooth muscle cells (VSMCs) are a primary contributor to the various stages of atherosclerotic plaque development, possessing great plasticity and special clonality.^93^ During the early stages of atherosclerosis, VSMCs lose their contractile phenotype and exhibit proliferation and migration, accompanied by a myofibroblast-like transformation, also referred to as “smooth muscle cell phenotypic switching”. This transformation allows the cells to become foam cells and secrete inflammatory factors. Subsequently, VSMCs adopt a fibroblast-like phenotype in a self-repair mechanism known as “reverse differentiation”, while still retaining some macrophage-like characteristics, such as phagocytic capability. In the murine vascular milieu, VSMCs initially form the fibrous cap and subsequently transition to a synthetic phenotype in the lesion core. This process involves the conversion of contractile proteins into extracellular matrix (ECM) components and distinct lipid metabolisms.^93,94^ Hypoxia augments the uptake of palmitate and low-density lipoprotein (LDL) in VSMCs.^95^ Excessive uptake of palmitate and LDL elevates cholesterol levels in the bloodstream, contributing to the development of atherosclerosis. Under hypoxic conditions, HIF-1α targets and upregulates the 3-hydroxy-3-methylglutaryl-coenzyme A (HMG-CoA) reductase gene to facilitate cholesterol synthesis in VSMCs.^96^ Moreover, the increased levels of HIF-1α in hypoxic VSMCs activate the transcription of low-density lipoprotein receptor-related protein (LRP1). The functional consequence of hypoxia on LRP1 protein expression is an increased uptake of cholesterol via aggregated low-density lipoprotein, leading to elevated cholesterol ester accumulation. Furthermore, immunohistochemistry reveals a co-localization of LRP1 and HIF-1α in vascular cells within advanced atherosclerotic plaques in human tissues.^97^ The reduced ATP-binding cassette sub-family A member 1 (ABCA1)-mediated cholesterol efflux observed in hypoxic conditions may be attributed to subcellular redistribution and decreased protein levels of ABCA1. This regulation could potentially be influenced by HIF-1α via interference with vesicular transport, affecting ABCA1 recycling or protein misfolding.^98^ Furthermore, the elevated HIF-1α resulting from hypoxia can also impact the proliferation and migration of VSMCs. Macrophage migration inhibitory factor (MIF), a multifunctional pleiotropic cytokine, is expressed and secreted by macrophages, ECs, and VSMCs in response to atherosclerotic stimuli. MIF demonstrates cytokine-like properties, influencing the proliferation and migration of VSMCs. Research has demonstrated the regulation of MIF by HIF-1α in VSMCs exposed to hypoxia.^99^

The ECs constitute the initial interface interfacing with blood, thereby enabling them to orchestrate vascular homeostasis. This is sustained via a myriad of mechanisms, encompassing the enforcement of anti-thrombotic function, recruitment of inflammatory mediators, and modulation of vascular tone by ECs.^100^ Alterations in these mechanisms occur under conditions of vascular hypoxia. In the nascent stages of atherosclerosis, non-hypoxic stimuli such as angiotensin II, TNF-α, or mildly oxidized low-density lipoprotein may assume paramount significance in eliciting the activation of HIF in ECs.^90^ Conversely, during the intermediate and advanced stages of atherosclerosis, hypoxic stimulation, through the activation of HIF-1α, confers upon ECs the ability to adapt to diminished metabolic requisites in a low-oxygen milieu.^101^ HIF-1α instigates profound transformations in ECs via three principal ways: VEGF, nitric oxide (NO), and ROS. The generation of VEGF, free radicals, and NO, coupled with the upregulation of platelet-derived growth factor (PDGF), promotes the progression of atherosclerosis.^89,102^ Acute hypoxia leads to the activation of ECs, liberating inflammatory mediators and growth factors, thereby promoting the adhesion of phagocytes (such as neutrophils and macrophages) to the vascular wall. In the context of chronic hypoxia, the transcription of genes such as Glut-1 (participating in transendothelial glucose transport), VEGF, and iNOS is induced, enabling cells to endure in a low-oxygen milieu.^103^ Furthermore, endothelial dysfunction resulting from endothelial cell injury is intricately linked to the initiation and progression of atherosclerosis. SIRT6, as a member of the Sirtuins family, partakes in various physiological and pathological processes, including DNA repair, anti-aging, and metabolism.^104^ Recent research indicates that in atherosclerosis, SIRT6 notably enhances vascular genesis and plaque hemorrhage. In vitro, SIRT6 overexpression impedes the degradation of HIF-1α via ubiquitination, thus fostering the invasive, migratory, proliferative, and tube-forming abilities of HUVECs under normoxic and hypoxic conditions. Interestingly, under distinct circumstances, SIRT6 exerts divergent roles in HUVECs’ functionality. On one the hand, in hypoxic conditions, SIRT6 facilitates vascular genesis within carotid artery plaques by mediating HIF-1α. Conversely, under oxidative stress, which constitutes another crucial pathological milieu in atherosclerosis, SIRT6, by means of removing the H6K3Ac modification at the promoter of the hydrogen peroxide scavenger enzyme, suppresses the expression of said enzyme at the transcriptional level. This, in turn, leads to the accumulation of ROS within cells, promoting endothelial injury in carotid arteries as well as neovascular damage within plaques, ultimately culminating in carotid artery plaque hemorrhage. In concert, the interaction of these two factors ultimately accentuates the instability of carotid artery plaques.^105^ In summary, this groundbreaking study corroborates the dualistic role of SIRT6 in different contexts, unraveling the intricate role of SIRT6 in atherosclerosis.

#### HIF in PAH

PAH is characterized by persistent pulmonary vascular constriction and proliferative and occlusive vascular remodeling. Its pathological underpinnings encompass media thickening, marked by increased EC proliferation and volumetric expansion, intimal dysregulation typified by EC dysfunction and apoptosis; perivascular inflammatory infiltration; in situ thrombosis; and ultimately augmented vascular stiffness, particularly within the distal pulmonary arteries.^106–108^ Recent research has revealed that even in the absence of endothelial denudation, hypoxia can incite pulmonary arterial constriction.^109^ Hypoxia-induced pulmonary vascular constriction represents a response to acute pulmonary oxygen insufficiency, undertaken with the aim of optimizing gas exchange. In contrast, chronic hypoxia precipitates pathological vascular remodeling, culminating in PAH.^110^

The predominant phenotypes of pulmonary artery smooth muscle cells (PASMCs) alter between proliferation, inflammation, and ECM generation, which exhibits great similarity with VSMCs in atherosclerosis.^111^ However, in contrast to atherosclerosis, the distinctive manifestations of VSMCs in PAH primarily involve electrophysiologic maladaptation and constriction. Under conditions of pulmonary alveolar hypoxia, mitochondrial sensors dynamically modulate ROS and redox coupling within PASMCs. This process inhibits potassium channels, leading to PASMCs depolarization. Additionally, it also activates calcium channels, elevating intracellular calcium levels, ultimately culminating in vasoconstriction.^112^ Furthermore, a reduction in the expression of voltagegated potassium channels (Kv), which is reliance on HIFs, also promotes remodeling by diminishing cellular apoptosis.^113–116^ PASMCs, in contrast to most cultured primary cells, exhibit elevated levels of HIF-1 under both normoxic and hypoxic conditions.^31^ Furthermore, in PASMCs, HIF subtypes not only regulate genes associated with cell proliferation and synthetic phenotypes but also play a role in the regulation of genes associated with vasoconstriction (Ca^2+^ modulation/ion channels), mitochondrial fragmentation, oxidative stress, and the renin-angiotensin-aldosterone system. Under hypoxic conditions, HIF-1 and HIF-2 govern the proliferation, migration, and apoptosis phenotypes of PASMCs via various pathways. For instance, HIF-1 activates the expression of miR-9-1 and miR-9-3, thereby promoting PASMC proliferation and phenotypic switching.^117^ HIF-1 mediates miR-322 expression, which in turn blocks the BMP-Smad signaling pathway, thus facilitating PASMC proliferation and migration.^118^ HIF-1-dependent miR-210 exerts a protective anti-apoptotic effect on PASMCs during hypoxia.^119^ Meanwhile, HIF-2α stimulates PAH-driven vascular remodeling and vasoconstriction by upregulating thrombospondin-1 during hypoxia.^120^ Epigenetics also plays a crucial role in regulating the VSMC phenotype in PAH, which will be further discussed in Section 2.2.

Pulmonary artery endothelial cells (PAECs) exhibit distinct phenotypes during the pathogenesis of PAH, including proliferation, migration, vascular angiogenesis, and/or endothelial-to-mesenchymal transition (EndoMT). HIF subtypes play a pivotal role in defining these phenotypes. For instance, HIF-1 mediates the upregulation of cyclin-dependent kinase inhibitor 1B (p27Kip1) and downregulation of cyclin-dependent kinase 4 (CDK4), leading to reduced proliferation and migration of hypoxic PAECs.^121^ Conversely, HIF-2, acting through miR-130/131, orchestrates the inhibition of the peroxisome proliferator-activated receptor gamma (PPARγ)/apelin signaling pathway, thereby enhancing expression of Oct-4 or FGF2, ultimately resulting in heightened proliferation of PAECs.^122,123^ In hypoxic PAECs, estrogen, mediated by estrogen receptor beta (ERβ), can upregulate PHD2, thereby promoting the degradation of HIF-1α and HIF-2α, ultimately attenuating the progression of hypoxic pulmonary arterial hypertension (HPAH).^124^

Furthermore, the interplay and differential effects of HIF-1 and HIF-2 in PAH are worth exploring. HIF-1α is predominantly expressed in PASMCs, HIF-2α is chiefly found in PAECs, and HIF-3α is primarily expressed in pulmonary fibroblasts.^125^ Mutations in HIF-2α have been shown to enhance PAH in both murine models and human patients,^126,127^ and HIF-2α may exert a significant influence on the pathogenesis of PAH, whereas HIF-1α is likely to mainly contribute to disease progression and persistence.^128^ In sum, HIF-2α emerges as a crucial driving factor in the early stages of the disease, with transitional interactions with HIF-1α.

Collectively, oxygen sensing assumes paramount importance in the vital activities of organisms. Extensive research has unveiled the pivotal role of the HIF pathway in the process of embryonic heart development, and its deficiency or excessive accumulation may lead to incomplete cardiac development, culminating in embryonic lethality. Myocardial regeneration has long been a sought-after goal in regenerative medicine and the connection between cellular pluripotency, hypoxia, and HIF activity has been established. Remarkably, studies have discerned that adult cardiomyocytes possess the ability to re-enter the cell cycle under extreme conditions, such as hypoxia. Hence, harnessing the potential of hypoxia may serve as a strategic avenue for inducing human cardiac regeneration. Furthermore, the cardinal significance of HIF-1α extends beyond cardiac regeneration, encompassing its active involvement in cardiovascular diseases. HIF-1α emerges as a significant contributor to the pathogenesis of conditions including atherosclerosis, PAH, and cardiomyopathy. During transient myocardial ischemia, IPC, I/R injury, and acute MI, HIF-1α exhibits a protective effect on the myocardium, ameliorating the detrimental consequences. However, the long-term sustained activation of HIF-1α exacerbates chronic heart failure, underscoring its dual role in cardiac pathophysiology.

## Multi-level regulatory signaling pathways and mechanisms of hypoxia signaling in cardiovascular diseases

### HIFs switch of hypoxia signaling in cardiovascular diseases

#### Classical “HIFs switch”

Due to the structural similarity of HIF-1α and HIF-2α, the common changes in energy metabolism induced by HIF-1/2α in cardiovascular cells under hypoxia determine the basis of HIFs’ response. However, given the different properties of HIF-1α and HIF-2α that still exist, in the interconnected, feedback space-time, HIF-1α and HIF-2α play a dominant role in their own specific time and place, which is called the "HIFs switch." The conventional and classical "HIFs switch" refers to the sequential substitution of dominant roles between HIF-1/2α isomers during hypoxia (including severity, phase [acute vs. chronic]), while the generalized ‘HIFs switch’ further includes the balanced succession of natural selection for HIF-αs and its associated molecules in longer time dimensions (Fig. 2a).^6^

First, the difference in structure (physicochemical properties) largely determines the difference between HIF-1α and HIF-2α participating in the reaction. A transactivation domain, N-TAD plays a crucial role in ensuring the target gene specificity of both HIF-1α and HIF-2α. The second transactivation domain (C-TAD) is conserved across isoforms and facilitates the activation of their shared target genes.^129^ The basic metabolic adaptation of mammal cells to hypoxia, which is also referred to as the Pasteur effect, is shifting from aerobic to anaerobic metabolism to raise glucose uptake, elevate glycosis, and generate ATP independently of oxygen alongside reduced mitochondrial oxidative phosphorylation (OXPHOS).^130^ HIF-1 governs glycolysis and pyruvate metabolism, while HIF-2 controls fatty acid metabolism (Fig. 2a).^10,131^ HIF-1 and HIF-2 collaborate to reprogram metabolic pathways, generating cellular energy when oxidative phosphorylation is hindered by limited oxygen availability. Furthermore, FIH-1 preferentially hydroxylates HIF-1α due to the greater proximity of valine to the hydroxylated asparagine (compared to the alanine of HIF-2α), while remaining active at lower oxygen tensions than the PHDs, thus suppressing HIF-1α transcriptional activity at certain moderate or intermediate hypoxic conditions and allowing HIF-2α still to be active.^1,132,133^ As the oxygen levels decrease, PHD first becomes inactive, allowing the expression of genes sensitive to N-TAD (ODDD or N-TAD-mediated transcription). Under severe hypoxia, both PHD and FIH become inactive, leading to the expression of genes sensitive to both N-TAD and C-TAD (N-TAD/C-TAD-mediated transcription).^134,135^ Specific to the level of transcriptional activity and its regulation, classically, during acute hypoxia, HIF-1α plays a dominant role, mainly inducing angiogenesis. In the subsequent chronic sustained hypoxia, HIF-2α plays a leading role, mainly making new blood vessels mature and stable (such as the recruitment or development of pericytes, and smooth muscle cells, etc.).^6,132^ HAF, miR-429, miR-155, and miR-200b have been observed to be participated in this process. However, unlike persistent stimuli, intermittent hypoxia triggers the activation of HIF-1α^136^ while suppressing HIF-2α^137^.

Furthermore, switching between isoform expressions is believed to be involved in the pathogenesis of numerous diseases and can potentially mediate the therapeutic effects.^138^ HIF-1α and HIF-2α facilitate cellular adaptation to acute hypoxia, but prolonged activation yields contrasting effects on redox state and proinflammatory pathways. Imbalances between these isoforms may contribute to chronic cardiac, vascular, and renal disorders.^139–141^ HIF-1α and HIF-2α demonstrate numerous synergistic aspects in chronic pathological processes of cardiovascular system. The dysregulation of oxygen-consuming cellular components leads to an abnormal increase in oxidative stress. This, in turn, triggers the activation of HIFs, which directly or indirectly mitigate oxygen consumption and the generation of ROS by mitochondria and peroxisomes.^142–144^ Both HIF-1α and HIF-2α facilitate autophagy, a lysosome-mediated degradation process that eliminates dysfunctional organelles (HIF-1α for mitophagy^145^, HIF-2α for pexophagy^146^). However, the interaction between HIF-1α and HIF-2α plays a crucial role in establishing the cellular redox state, while simultaneously exerting opposing effects on inflammation and fibrosis across various organs such as the heart, kidney, vasculature, and adipose tissue (Table 1).^147–149^ The impact of HIF-1α signaling on enhancing adaptation to acute hypoxia is rapid and transient. In contrast, sustained activation of HIF-1α signaling leads to the promotion of oxidative stress, inflammation (via iNOS activation, M1 macrophage polarization, and proinflammatory cytokine release), and fibrosis (through profibrotic chemokines and collagen deposition).^149–152^ Alternatively, HIF-2α can mitigate oxidative stress by promoting pexophagy and activating antioxidant enzyme genes.^153^ It also opposes inflammation and fibrosis (promoting collagen matrix degradation).^149,154^ Considering their ability to inhibit HIF-1α and activate HIF-2α, SIRT1 activators (e.g., SLGT2 inhibitors, resveratrol) and selective PHD inhibitors (e.g., cobalt, roxadustat) are extensively studied and used to treat chronic cardiovascular and renal diseases.^139^ Collectively, the current primary treatment strategy for addressing spatiotemporal specificity of the "HIFs switch" is as follows: during the acute phase, activate HIF-αs effectively; during the chronic phase, prioritize the predominance of HIF-2α efficacy while maintaining appropriate HIF-1α activity.

**Table 1:** The interplay between HIF-1α and HIF-2α in different cardiovascular and kidney diseases

Diseases	HIF-1α	HIF-2α
Ischemic and Failing Heart	***Acute phase:*** ● HIF-1α inhibition → insufficient vascularization, decompensation of acute pressure overload or ischemic conditions.^619^ ***Chronic phase:*** ● Prolonged HIF-1α stimulation → decline of cardiac function.^82^ ● HIF-1α-induced shift to glycolysis → mitochondrial impairment (with oxidative stress elevation), lipid accumulation, myocardial hypertrophy and inflammation → poor adenosine triphosphate generation and dysregulated myocardial contraction.^144,620,621^ ● Genetic upregulation of HIF-1α → cardiomyopathy.^80^	● HIF-2α activation (in the media and endothelium of coronary and renal arteries) → protection of tissue injury.^622,623^ ● HIF-2α upregulation (in myocardium) → adaptive ventricular remodeling.^624^ ● HIF-2α activation (in side population (SP) cells, a cardiac progenitor cell population) → protection of cardiomyocytes.^625^ ● Decreased HIF-2α (the whole heart sample)→ upregulation of cardiac inflammasome.^626^
Atherosclerosis	● Elevated intravascular pressures (or sheer stress) and the infiltration of inflammatory cells into the middle layer → HIF-1α activation.^627^ ● Increased expression of HIF-1α (in vascular smooth muscle, endothelial cells and macrophages (dominant M1 phenotype)) → development of neointimal proliferation, medial hypertrophy, and stimulation of proinflammatory signalings → advancement of atherosclerosis.^95,628,629^ ● HIF-1α activation (in smooth muscle cells) → cholesterol accumulation, and cell proliferation and migration.^27^ ● Vascular wall hypoxia → HIF-1α activation → arterial thrombus formation.^630^	● Potential HIF-2α deficiency: oxidized lipoproteins → HIF-2α inhibition under hypoxia.^631^ ● Enhancement of HIF-2α → inhibition of neointimal proliferation and atherosclerosis.^92^
Cardiac hypertrophy	● On set of cardiac hypertrophy: myocardial HIF-1 activation → fatty acid oxidation to glycolysis shift.^6,632^ ● Myocardial HIF-1 activation → intracellular Ca^2+^ regulation → pathological hypertrophy.^633^	● Global loss of HIF-2α → mitochondrial metabolism dysfunction and elevated ROS production → cardiac hypertrophy.^153^ ● HIF-2α activation in adipocytes → regulation of lipid metabolism and inflammatory signaling → cardiac hypertrophy.^634^
Ventricular arrhythmia	***Simple ischemia/hypoxia:*** ● Myocardial HIF-1α activation → elevated cytoplasmic influx of Na^+^ and Ca^2+^, and decreased efflux of K^+^ → reduced transmembrane potential *E*m → dysregulated cardiac contraction and impaired myocardial viability.^6^ ***Ischemia-reperfusion:*** ● Interaction between myocardial HIF-1α and NF-κB → reduced myocardial ion channels → arrhythmogenesis.^635,636^	● *Unknown*
Pulmonary arterial hypertension (PAH)	***Overall:*** ● HIF-1α activation → advancement and persistence of PAH.^128^ ***PASMCs (pulmonary artery smooth muscle cells):*** ● HIF-1α activation → increased miR-9-1/9-3 → promoting PASMC proliferation and phenotypic switching.^117^ ● HIF-1α activation → increased miR-322 → blocked BMP-Smad pathway → facilitated PASMC proliferation and migration.^118^ ● HIF-1α activation → increased miR-210 → reduced apoptosis.^119^ ● Reactive oxygen species (ROS) → HIF-1α activation → increased cytoplasmic K^+^, elevated Ca^2+^ influx, and intracellular alkaline shift → reduced apoptosis, cell depolarization, and vasoconstriction.^27^ ***PAECs (pulmonary artery endothelial cells):*** ● HIF-1α activation → increased p27 and reduced CDK4 → reduced PAECs proliferation and migration.^121^	● **Overall:** HIF-2α → on the onset of PAH.^128^ ● HIF-2α activation (in PASMCs) → increased vessel stiffness, enhanced vasoconstriction, and compromised endothelial barrier.^120,637,638^ ● Hypoxia → HIF-2α activation → increased TSP1 → closed pulmonary Kv channels → stimulate vascular remodeling and vasoconstriction (in PASMCs).^120^ ● Estrogen → increased PHD2 → promoted HIF-2α degradation → attenuated hypoxic PAH progression (in PAECs).^124^ ● Hypoxia → HIF-2α activation → increased miR-130/131 → reduced PPARγ → increased Oct-4 → cell proliferation (in PAECs or PASMCs).^122,123^
Cardiac fibrosis	● TGF-β → SMAD2/3 → PHD2 inhibition → myocardial HIF-1α activation → profibrotic process.^639^ ● Damage-associated molecular patterns (DAMPs) → NF-κB signaling → myocardial HIF-1α activation → proinflammatory signals.^640,641^ ● Depletion of HIF-1α in cardiac fibroblasts (after acute ischemic injury) → elevated ROS → excessive fibrosis.^642^	● Myocardial HIF-2α → long non-coding RNA (lncRNA) *Neat1* in small extracellular vesicles → fibroblast activities.^643^
Aneurysm	● Excessive cytoplasmic HIF-1α accumulation (in vascular smooth muscle cells) → cell apoptosis.^27^ ● HIF-1α depletion → reduced formation of elastic fibers (in vascular smooth muscle cells).^644^ ● TGF-β → elevated SMAD3 → SMAD3-HIF-1 complex → VEGF-A → anti-apoptosis (in vascular smooth muscle cells).^645,646^	● *Unknown*
Vascular calcification	● HIF-1α may be involved in a process similar to the early stages of ossification in vascular calcification.^138^ ● inorganic phosphate, advanced glycation end products, etc → interaction of HIF-1α stablizaion, unfettered ROS and RUNX2 → osteochondrogenic response (in vascular smooth muscle cells).^27^	● HIF-2α may be involved in a process similar to the later stages of ossification in vascular calcification.^138^
Kidney disease^a^	***Acute phase:*** ● Silencing HIF-1α during low-oxygen tension → inhibition of angiogenesis and tissue repair → renal tubular epithelial cell necrosis.^647–649^ ***Chronic phase:*** ● Prolonged elevation of HIF-1α (in renal tubular epithelial cells) → epithelial to mesenchymal transition.^650^ ● Sustained HIF-1α signaling (in the glomerulus and renal tubules) → proinflammatory and profibrotic pathways.^651–654^	***Acute phase:*** ● Endothelial HIF-2α upregulation → a comparable protective effect on the kidneys.^623^ ***Chronic phase:*** ● Chronic kidney disease: HIF-2α deficiency → anemia, increased inflammatory and angiogenic markers.^148,655–657^ ● Diabetic kidney: hyperglycemia and advanced glycation end products → promotion of HIF-1α transcription in glomerular and renal tubular cells and inhibition of SIRT1 activity.^658–660^

^a^The filtration function of the kidney is an important indicator reflecting the circulating blood volume, and the urine output of shock patients is a direct indication of tissue perfusion. Therefore, kidney disease is closely related to circulatory system disease in many cases and will be taken into overall consideration (such as diabetic nephropathy and heart failure, renal hypertension, etc.)

#### "HIFs switch" in a broader sense

Additionally, for "HIFs switch" in a broader sense, natural selection of high altitude is a long-term factor. The plateau is one of the hypoxic environments that mammals can be exposed to under physiological conditions, where hypobaric hypoxia is a significant and defining feature. For lowlanders, two key high-altitude factors, hypoxia (6% O_2_) and low temperature (0 °C), enhance the HIF-1α level to promote the expression of the transferrin gene (the enhancer region contains HIF-1α binding sites), thereby inducing hypercoagulability by enhancing thrombin and FXIIa. Notably, anti-transferrin antibody, transferrin knockdown, and peptide interference treatment almost completely suppressed hypothermia- and hypoxia-induced hypercoagulability.^155^ Furthermore, HIF-2α may serve as a beneficial short-term adaptation mechanism at high altitudes, but excessive activation of HIF-2α can lead to chronic mountain sickness, which can be fatal and impede reproductive capabilities. Moreover, certain mutations resulting in heightened HIF-2α expression are linked to elevated risk of hypertension and stroke at low altitudes, exhibiting symptoms akin to mountain sickness.^156^ However, high-altitude populations undergoing an evolutionary selection of HIF-2α variants could lessen the adverse fitness effects of excessive RBC production.^156,157^ Sizeable human populations have successfully settled at elevations exceeding 2500 m in three distinct geographic areas worldwide, namely the Ethiopian Highlands, the Andean Altiplano, and the Tibetan Plateau.^158–160^ Under the long-term pressure choice, the hypoxia response pathway (e.g., HIF-α and PHD) of residents in plateau area has changed adaptively, which is natural selection-induced "HIFs switch." Among them, in addition to the reactive metabolic reprogramming of cells to hypoxia, the body’s natural selection for hemoglobin (Hb) level and its oxygen-carrying capacity and regulation of vasomotor (e.g., NO) is also particularly important (Fig. 2b).

The Qinghai-Tibetan plateau, the largest high-altitude region (including Himalayas), has been inhabited by humans for around 25–30,000 years,^161^ and a substantial portion of Tibetans possess the *EPAS1* mutation (encoding HIF-2α) enhancing oxygen transport.^156,162–165^ Compared to lowlanders, Tibetan-enriched *EPAS1* variants are linked to lower circulating Hb levels, reduced susceptibility to hypoxic pulmonary vasoconstriction, and increased glycolysis as indicated by elevated circulating lactate.^159^ Indeed, Sherpas at high altitudes of Nepal’s Himalayas also exhibited elevated lactate dehydrogenase activity in their skeletal muscles compared to lowlanders, resulting in reduced accumulation of glycolytic intermediates.^166^ Furthermore in Tibetans, a relative switch away from fatty acid oxidation (FAO) to glucose metabolism has been associated with the single-nucleotide polymorphisms (SNPs) of *PPARA* (encoding PPARα).^164^ HIF-1 suppresses *PPARA* expression in mice under hypoxic conditions,^167^ while the antidiabetic drug tesaglitazar, which activates *PPARA*, led to reduced hemoglobin levels in human clinical trials.^168^ This switch could suppress FAO with increased circulating fatty acids,^169^ could enhance mitochondrial coupling by modifying tricarboxylic acid (TCA) cycle intermediates, and ultimately improve oxygen utilization efficiency.^166^ Accordingly, similar decreased oxidative metabolism in cardiac and skeletal muscle, including a decline in FAO capacity, and an increase in glycolysis, was also observed in lowlanders as they ascended to high altitudes,^159,166^ potentially influenced by epigenetic mechanisms such as methylation,^170^ which may induce beneficial, heritable features in a long run.^171,172^ Another observed positive selection is *EGLN1*, encoding PHD2.^164^ *EGLN1* mutation is supposed to be a loss of function variation, inducing the suppression of the HIF-αs’ degradation.^173^ By binding to the HSP90 cochaperone p23, PHD2 can be efficiently recruited to the HSP90 pathway, thereby facilitating the hydroxylation of HIF-α. The Tibetan PHD2 haplotype (D4E/C127S) significantly reduces the interaction between PHD2 and p23, leading to compromised down-regulation of the HIF pathway by PHD2. Furthermore, certain variants in the *GCH1* gene (encoding GTP cyclohydrolase 1(GTPCH)), responsible for stabilizing NO synthase activity, have been identified as targets of selection in Tibetans.^174,175^ *GCH1* was observed to be regulated by HIF-1/GTPCH/BH4 axis, which driving NO production,^176^ and these *GCH1* variants are linked to increased levels of circulating NO in Tibetans, contributing to improved pulmonary perfusion and protection against pulmonary hypertension at high altitudes.^174,177,178^ Higher levels of circulating NO metabolites in Tibetans are linked to increased blood flow in the limbs and regulation of cardiac and skeletal muscle metabolism in hypoxic conditions.^179–181^ Collectively, thanks to these plateau acclimatization, Tibetans do not experience altitude sickness and have lower levels of hemoglobin, adequate for reduced oxygen levels (Fig. 2b). They possess intricate blood vessels,^182^ lower infant mortality rates,^183^ and higher birth weights.^184^

Similarly, *EGLN1* and *EPAS1*, both identified as crucial genes for adaptation, have been found in Andeans who have inhabited the Andean Altiplano for around 11,500 years or more (Fig. 2b).^165,185–187^ However, Andeans adapt to high-altitude environments by significantly increasing their RBC and Hb levels, surpassing both lowlanders and Tibetans.^188,189^ Additionally, *PRKAA1* and *EDNRA*, related to infant birth weight, are also included in the positive selection profiles of Andeans.^190^ *PRKAA1* encodes the α1 catalytic subunit of AMP-activated protein kinase (AMPK) which is a cellular energy detector. A variant in *PRKAA1* has been associated with greater uterine artery diameter, improved cardiometabolic homeostasis, and its encoding AMPK is essential for transcriptional activity of HIF-1, whose transactivations are critical for embryonic vascularization and development, as well as closely connected with physiological adaptations to hypoxia and pregnancy.^190–192^ *EDNRA* encodes the *EDN1* receptor primarily expressed in vascular smooth muscle cells, which binds with the peptide ET-1, a potent vasoconstrictor associated with vascular homeostasis. Both hypoxia-induced HIF-1 and HIF-2 seems to possess the ability to elevate the ET-1 expression. Existing studies have shown that ET-1 in the blood of pregnant human women is spontaneously reduced to prevent intrauterine growth restriction (IUGR) due to vascular restriction and potential hypoxia.^193^ Also, animal studies have revealed a notable reduction in the expression of the EDNRA protein in the uteroplacental vascular bed, which could effectively prevent hypoxia-induced IUGR in rats.^194,195^ Thus, the *EDNRA* mutation may further reduce the interaction between ET-1 and its receptors in the bloodstream, leading to a further decrease in its vasoconstrictive effects, thereby protecting against altitude-induced IUGR. However, deeper mechanisms are yet to be revealed. Additonal genes related to cardiovascular development and cardiometabolic function, such as *NOS2A*^165^ and *NOS3*^196^ (encoding iNOS), and *BRINP3*,^197^ have also shown significant positive selection. *NOS2A*, encoding iNOS, a downstream of HIF-1, has been shown as a cardioprotector for the ischemic myocardium.^198,199^ Additionally, Ethiopians, who have been living on the Semien Plateau of Ethiopia for approximately 70,000 years, exhibit a significant signal of selection in the *BHLHE41* gene (encoding basic helix-loop-helix family member E41, also referred to as *DEC2* or *SHARP1*) (Fig. 2b).^200^ BHLHE41, a known regulator of circadian rhythm,^201^ also plays a role in the HIF pathway. It is transcriptionally activated by HIF-1α but acts to repress HIF targets by facilitating increased degradation of both HIF-1/2α.^202–204^ Furthermore, the *THRB* and *ARNT2* genes of Ethiopians have relatively significant changes.^205^ *ARNT2*, which encodes HIF-1β, plays a direct role in the HIF-1 pathway, while *THRB* is essential for HIF expression specifically in hepatic cells.^206^ *EPAS1*, *EGLN1*, and *PPARA* are genetic signals that have been identified in various highland populations, including Ethiopians.^205,207^ Ethiopians have higher hemoglobin concentrations, but not to the same extent as Andeans,^189^ while the relationship between adaptive responses of these variants and Hb remains umbiguous.

In summary, human populations living in high-altitude environments have adapted through natural selection. Key genes, including *EPAS1*, *EGLN1*, and *PPARA*, play important roles in oxygen transport, metabolism, and vascular regulation. Tibetans have lower hemoglobin levels and improved oxygen utilization, while Andeans have higher RBC and hemoglobin levels. Other genes like *PRKAA1*, *EDNRA*, *NOS2A*, *BRINP3*, *BHLHE41*, *THRB*, and *ARNT2* are also involved in cardiovascular development and metabolic function. These genetic adaptations provide insights into human evolution and potential treatments for altitude-related health issues.

### Epigenetic regulation of hypoxia signaling in cardiovascular diseases

Epigenetic regulation represents a pivotal genetic regulatory phenomenon that operates autonomously from the underlying genomic DNA sequence, exerting a momentous influence on the intricate orchestration of gene expression.^208–210^ This intricate process encompasses a diverse array of mechanisms, with prominence placed on the post-translational modifications of histones, DNA methylation alterations, and post-transcriptional regulation of RNA.^208,211^ Hypoxia denotes a diminution in the provision of oxygen to the body or specific tissues, which disrupts normal cellular processes. Diverse factors contribute to the onset of hypoxia, encompassing environmental circumstances such as high altitude and rigorous physical exertion, as well as certain pathological conditions. Within the realm of diseases, hypoxia assumes a pivotal pathological hallmark in both cancer and cardiovascular ailments, predominantly attributable to impaired perfusion of local tissues. Formerly, attention was predominantly focused on identifying genetic variations that influence human physiological adaptation and maladaptation to hypoxic conditions.^212^ Nonetheless, emerging evidence has unveiled that, in the context of cardiovascular diseases, certain hypoxia-induced genes are subject to epigenetic regulation.

#### Histone modifications

Post-translational modifications (PTMs) of histones primarily regulate the transcriptional processes of genes by influencing the positioning and compaction of nucleosomes.^213,214^ Throughout crucial biological processes like DNA replication, transcription, and repair, dynamic adjustments occur in the spatial arrangement and tightness of nucleosomes. Typically, gene transcription is activated in regions characterized by a relaxed disposition of nucleosomes, whereas regions exhibiting dense packing of nucleosomes tend to repress transcription.^215^ Common PTMs of histones include methylation, acetylation, phosphorylation, ubiquitination, small ubiquitin-related modifie, lactylation, ADP-ribosylation, crotonylation, citrullination, proline isomerization, propionylation, among others.^210,216–218^ These modification can occur in diverse combinations, yielding a myriad of biological functions. Upon histone modification, the chromatin architecture undergoes changes, subsequently affecting the interactions between DNA and histones. This, in turn, regulates gene transcriptional expression.

**Fig. 3:**
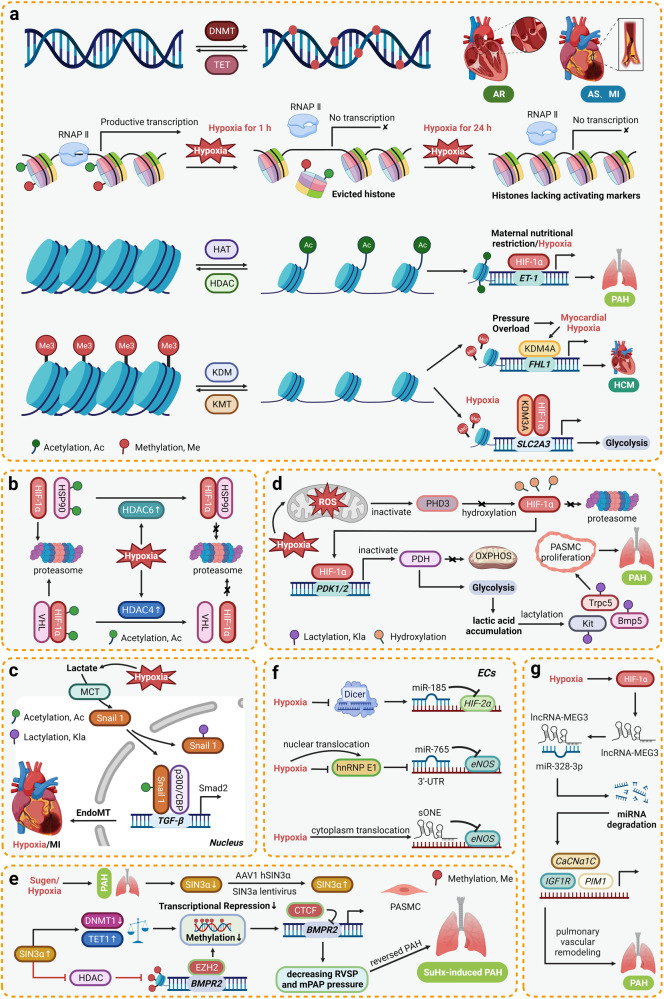
Epigenetic control of hypoxia signaling in cardiovascular diseases. **a** Post-translational modifications of histones and DNA methylation modifications. **b** The crucial role of Hsp90’s chaperone function in the regulation of HIF-1α stability. **c** The functional impact of lactylation modification in myocardial infarction. **d** The functional impact of lactylation modification in PAH. **e** The functional impact of DNA methylation modifications in PAH. **f** The influence of non-coding RNA on endothelial function under conditions of hypoxia or local hemodynamic disturbance. **g** The functional impact of long non-coding RNA in PAH. (*Created with BioRender.com*). Abbreviations: 3′-UTR 3′ Untranslated Region, AAV1 adeno-associated virus serotype 1, AR aortic regurgitation, Bmp5 bone morphogenetic protein 5, BMPR2 bone morphogenetic protein receptor type 2, CaCNα1C L-type calcium channel-α 1C, CTCF CCCTC-binding factor, DNMT DNA methyltransferase, ECs endothelial cells, EndoMT endothelial-to-mesenchymal transition, eNOS endothelial nitric oxide synthase, EZH2 enhancer of zeste homolog 2, FHL1 four and a half LIM domains 1, HAT histone acetyltransferase, HCM hypertrophic cardiomyopathy, HDAC histone deacetylase, hnRNP E1 heterogeneous nuclear ribonucleoprotein E1, IGF1R insulin-like growth factor 1 receptor, KDM lysine demethylase, Kit stem cell factor receptor, KMT lysine methyltransferase, lncRNA-MEG3 long non-coding RNA Maternally Expressed Gene 3, MCT monocarboxylate transporter, MI myocardial infarction, miR-185 microRNA-185, miR-328-3p microRNA-328-3p, miR-765 microRNA-765, mPAP mean pulmonary arterial pressure, PAH pulmonary arterial hypertension, PASMC pulmonary artery smooth muscle cell, PIM1 proviral integration site for moloney murine leukemia virus 1, RNAP II RNA polymerase II, ROS reactive oxygen species, RVSP right ventricular systolic pressure, SIN3α switch-insensitive 3a, SLC2A3 solute carrier family 2 member 3, Smad2 mothers against decapentaplegic homolog 2, Snail1 snail family transcriptional repressor 1, SuHx sugen/hypoxia, TET ten-eleven translocation, TGF-β transforming growth factor-β, Trpc5 transient receptor potential cation channel subfamily c member 5

Histone PTMs are implicated in various aspects of cardiovascular diseases, including atherosclerosis and PAH, specifically the response of ECs to hypoxic signals. Exposure of ECs to short-term (1 h) or long-term (24 h) hypoxia conditions leads to endothelial dysfunction, partly due to reduced expression of endothelial nitric oxide synthase (eNOS), accompanied by significant decreases in acetylation and methylation levels of histones at the proximal promoter of eNOS.^219–222^ Mechanistically, during acute hypoxia (1 h), histones H3 and H4 are rapidly evicted from the eNOS proximal promoter. Such eviction reduces the levels of H3K9ac, H4K12ac, and H3K4me (marks essential for eNOS transcriptional activity) near the eNOS proximal promoter site and prevents the binding of RNA polymerase II (Pol II) to the eNOS proximal promoter, thereby suppressing eNOS transcription. After a prolonged period of hypoxia (24 h), the evicted histones are reincorporated at the eNOS promoter, but they lack the necessary marks for transcriptional activity, such as acetylation or methylation. At this stage, chromatin architecture becomes closed, preventing the binding of Pol II to the eNOS proximal promoter, leading to reduced eNOS transcription.^219^ Interestingly, this study discovered that the decreased expression of eNOS in ECs under hypoxic conditions is not primarily due to the deacetylation activity of histone deacetylases (HDACs), but rather through histone eviction, reducing the chromatin accessibility of the eNOS proximal promoter and ultimately inhibiting eNOS transcription (Fig. 3a). Upon reoxygenation of ECs, the ATP-dependent chromatin remodeler SMARCA4/BRG1 first associates with histones by recognizing acetylated lysine residues on the histone tails. It then utilizes ATP hydrolysis to provide energy for chromatin remodeling and alteration, thereby facilitating the restoration of eNOS.^219^ In summary, histone PTMs are essential for maintaining the transcriptional activity of eNOS in ECs.

PAH is a severe disease that can lead to right heart failure and even death, with hypoxia being a common etiological factor.^223^ HPAH is characterized by increased pulmonary artery pressure and hypoxia-induced pulmonary vascular remodeling (HPVR).^224,225^ The occurrence and development of PAH are closely associated with epigenetic factors. In a study conducted on newborn rats with intrauterine growth retardation (IUGR), maternal nutritional restriction and hypoxic conditions resulted in a significant increase in histone acetylation levels within the *ET-1* gene promoter of pulmonary vascular endothelial cells.^226^ This increase facilitated the binding of HIF-1α to the promoter, subsequently enhancing the expression of ET-1 in ECs (Fig. 3a). These acetylation changes may render the IUGR rats more sensitive to hypoxia in later life, thereby increasing the risk of developing severe PAH.

Histone lysine methylation is dynamically regulated by lysine methyltransferases (KMTs) and lysine demethylases (KDMs). The KDMs family includes lysine-specific demethylases (LSDs) and jumonji-C domain-containing histone lysine demethylases (JmjC-KDMs).^227^ During the pathological process of cardiac hypertrophy, significant changes occur in the levels of histone lysine methylation. These changes can be directly influenced by altering the activity or expression levels of JmjC-KDMs or indirectly mediated through HIF. It is currently known that certain JmjC-KDMs are direct targets of HIF, and their levels are upregulated during hypoxia. These include KDM2B, KDM3A, KDM4B, KDM4C, KDM5B, KDM5C, and the recently discovered KDM6B.^228–234^ It is worth noting that KDM4C and KDM6B have been demonstrated to interact with HIF and potentially exert synergistic activating effects.^235,236^ Cardiac hypoxia can occur due to cardiac ischemia, systemic hypoxia, or anemia, and it is typically a feature of cardiac hypertrophy and a key factor in the progression to heart failure.^237–239^ Numerous studies have shown that local or chronic hypoxia in cardiovascular diseases can affect the levels and activity of JmjC-KDMs through HIF, thereby influencing histone methylation status. Cardiac hypertrophy is induced by pressure overload stimulation, and prolonged pressure overload increases myocardial oxygen demand, leading to myocardial hypoxia.^240^ It has been reported that KDM4A protein levels are significantly upregulated in patients with hypertrophic cardiomyopathy. Mice with cardiac-specific overexpression of KDM4A exacerbate cardiac hypertrophy following pressure overload induced by TAC, while cardiac-specific knockout of KDM4A attenuates the hypertrophic response.^241^ The elevation levels of FHL1, a mechanobiological stress sensor implicated in cardiac hypertrophy development,^242^ have been found to be due to the binding of KDM4A to the FHL1 promoter, resulting in a decrease in H3K9 trimethylation levels and activates the transcription of FHL1 (Fig. 3a).^241^ In addition, under hypoxic conditions, KDM3A is recruited to the distal enhancer region of the solute carrier family 2A3 (*SLC2A3*) gene in a HIF-1-dependent manner. This recruitment leads to the demethylation of inhibitory histone modification H3K9me2 at the enhancer site, promoting increased expression of the *SLC2A3* gene. Consequently, it enhances the glucose uptake capacity of ECs under hypoxic conditions, providing more substrate for anaerobic glycolysis (Fig. 3a).^243^ Overall, this discovery provides a novel research perspective for gaining a deeper understanding of how ECs adapt to a hypoxic environment.

#### Non-histone modification

Histone acetylation plays a crucial role in DNA chromatin structure and gene transcription regulation. Non-histone acetylation has been demonstrated to regulate protein function and stability. The reversible acetylation of both histone and non-histone proteins at lysine residues is controlled by histone deacetylases (HDACs) and histone acetyltransferases (HATs).^244,245^ Apart from hydroxylation modifications, HIF-1α can undergo reversible lysine acetylation as a post-translational modification.^246–248^ Acetylation modification of HIF-1α enhances its interaction with von Hippel-Lindau protein (pVHL), facilitating the degradation of HIF-1α.^246^ Angiogenesis is crucial for the recovery of cardiac and circulatory system function after MI. Insufficient angiogenesis can hinder the restoration following ischemia and may lead to heart failure post MI.^249^ Regardless of whether under normoxic or hypoxic conditions, the chaperone function of heat shock protein 90 (Hsp90) can protect its target proteins, including HIF-1α, from degradation.^250^ Acetylation of Hsp90 leads to its dissociation from the target proteins, facilitating their degradation. Under hypoxic conditions, HDAC4 and HDAC6 are significantly upregulated. The increase in HDAC6 leads to a significant decrease in the acetylation level of Hsp90, thereby promoting the binding of Hsp90 to HIF-1α and protecting HIF-1α from proteasomal degradation.^248^ However, increasing HDAC4 does not reduce the acetylation level of Hsp90 nor disrupt the interaction between HIF-1α and Hsp90. Instead, it directly diminishes the acetylation level of HIF-1α protein, stabilizing HIF-1α (Fig. 3b).^251^

MI is a common complication caused by sustained ischemia in the heart or coronary arteries.^252^ Cardiac fibrosis is a pathological process involving ECM remodeling and activation of fibroblasts, which occurs in most cardiac injuries including MI. Early cardiac fibrosis can prevent the rupture of infarcted cardiac tissue during MI. However, persistent cardiac fibrosis is often associated with poor prognosis in patients, ultimately leading to heart failure.^253,254^ Lactate, a widely present intermediate product of glycolysis, was previously considered a metabolic byproduct. However, as clinical research has progressed, it has been discovered that high levels of lactate are positively correlated with prognosis and mortality rates in patients with heart disease.^255^ The study by Zhang et al.^218^ discovered that lactate derived from glycolysis can directly modify histones by adding lactyl groups to lysine residues, resulting in a modification known as “lactylation”. Similar to other epigenetic modifications, lactylation can modify histones to alter the spatial configuration of chromatin, affecting DNA accessibility and regulating the expression of corresponding genes. Importantly, the degree of lactylation is closely linked to local lactate concentration, bridging the gap between epigenetics and metabolic reprogramming. Furthermore, previous studies have found that cardiac hypoxia following MI promotes EndoMT, which plays a significant role in cardiac fibrosis. Inhibition of EndoMT can alleviate cardiac fibrosis.^256,257^ Transforming growth factor-β (TGF-β) is believed to play a crucial role in fibrosis associated with EndoMT in various cardiovascular diseases.^257,258^ A discovery was made indicating that lactate promotes cardiac fibrosis and exacerbates cardiac dysfunction following MI by promoting EndoMT.^259^ Under hypoxic conditions, lactate activates the TGF-β/Smad2 signaling pathway in ECs to facilitate EndoMT. Mechanistically, lactate transported into the cells through monocarboxylate transporters (MCTs), which are responsible for lactate transport, can induce nuclear translocation and lactylation of Snail1, a transcription factor involved in TGF-β signaling. Additionally, Snail1 can interact with transcription coactivators (p300/CBP) and induce acetylation modification of Snail1, ultimately activating the TGF-β/Smad2 signaling pathway to regulate EndoMT (Fig. 3c). Inhibiting Snail1 can reduce lactate-induced EndoMT and activation of the TGF-β/Smad2 pathway under hypoxia/MI conditions, thus improving cardiac dysfunction following MI. In conclusion, this discovery provides novel insights into the role of lactate in myocardial fibrosis and cardiac dysfunction. Recent studies have also discovered that lactylation mediates the transition of macrophages from the M1 pro-inflammatory phenotype to the M2 anti-inflammatory phenotype, and a similar transition has been observed in atherosclerosis. This suggests that lactylation-mediated macrophage polarization plays a significant role in chronic inflammatory diseases.^260–262^ One prominent characteristic of PAH is HPVR, primarily manifested by progressive proliferation of PASMCs.^263,264^ In recent years, an increasing body of research has indicated a close association between glycolytic shift and the pathogenesis of PAH.^265,266^ However, the mechanisms underlying the enhancement of glycolysis during PAH development and how increased glycolysis promotes HPVR in PAH remain unknown. Chen et al.^101,267,268^ conducted a study and found that hypoxia-induced mitochondrial reactive oxygen species (mROS) inactivate the hydroxylation of prolyl hydroxylase 3 (PHD3), thereby enhancing the stability of HIF-1α. Under hypoxic conditions, the accumulation of HIF-1α promotes the glycolytic shift in PASMCs through the phosphorylation of pyruvate dehydrogenase (PDH) mediated by pyruvate dehydrogenase kinase 1 (PDK1) and PDK2. This, in turn, leads to increased lactate production and lactylation modification of histones. Further investigations revealed that lactate accumulation promotes lactylation modification of downstream targets of HIF-1α associated with the proliferative phenotype, such as Bmp5, Trpc5, and Kit, thus facilitating PASMC proliferation (Fig. 3d). Knocking down PDK1/PDK2 reduces lactate accumulation, lactylation modification levels, and PASMC proliferation. These findings highlight the functional role of lactylation modification in PAH and provide proof-of-concept for the potential therapeutic approach of controlling lactate levels to counteract vascular remodeling. Therefore, modulating lactylation modification of histones and non-histone proteins may serve as a powerful adjunctive therapeutic target to influence the epigenetic landscape of cardiovascular diseases.

#### DNA methylation modifications

The most well-known epigenetic DNA modification is 5-cytosine methylation, which commonly occurs in clusters known as “CpG islands” in gene promoter regions.^211,269^ This modification is crucial for proper gene expression, transposon silencing, alternative splicing, and genome stability.^270,271^ Several studies have linked DNA methylation to cardiovascular diseases. Cardiovascular risk factors such as smoking, dyslipidemia, low dietary folate intake, and elevated plasma homocysteine levels can lead to dysregulation of DNA methylation.

Transient tissue fibrosis is a normal response to injury during the wound healing process. However, pathological fibrosis refers to the continuous, non-dissolving deposition of ECM and progressive tissue remodeling, which is detrimental to the function of the heart.^272^ Several stimulating factors can lead to pathological fibrosis of the heart, including ischemia, inflammation, pressure overload (such as hypertensive heart disease and aortic stenosis), and volume overload (such as mitral valve or aortic regurgitation).^273^ All of these stimuli share a common characteristic, namely tissue hypoxia. This hypoxia can occur directly due to insufficient oxygen supply or indirectly due to increased oxygen consumption by infiltrating inflammatory cells and activated resident cells. Prolonged local tissue hypoxia can result in abnormal remodeling of the ventricle and fibrosis of the heart.^274–276^ Research has revealed that DNA methylation plays a significant role in determining cellular response to chronic hypoxia and the progression of cardiac fibrosis. Chronic hypoxia leads to a notable upregulation of DNA methyltransferase 1 (DNMT1) and DNMT3B expression, and this upregulation is mediated by HIF-1α. Consequently, there is a substantial increase in overall DNA methylation levels within the cardiac fibroblast genome (Fig. 3a).^277^ To further investigate the impact of DNMT on cardiac fibrosis, researchers transiently knocked down DNMT3B using siRNA transfection, and the results revealed a significant decrease in the expression levels of fibrosis markers, collagen protein, and α-smooth muscle actin. This study provides potential therapeutic targets for the treatment of myocardial fibrosis.

Over the past decade, a wealth of evidence has revealed that epigenetic mechanisms, such as DNA methylation modifications, play a pivotal role in the regulation of gene expression in HPAH^278–282^ Indeed, DNA methylation may induce chromatin structural alterations via the recruitment of methyl CpG-binding proteins (MeCP) and HDACs. These structural changes impede the binding of DNA to transcription factors (TFs) and, subsequently, transcription initiations.^282–284^ Aberrant expression of DNMT and MeCP2 can disrupt DNA methylation levels, consequently giving rise to pathological phenotypes.^211,285–287^ A recent study has elucidated a strong correlation between hypermethylation of the bone morphogenetic protein receptor type 2 (BMPR2) promoter and both the downregulation of BMPR2 and the progression of PAH.^288^ Switch-insensitive 3a (SIN3a) is an established transcriptional regulator that plays a significant role in gene expression modulation. Novel findings indicate that in Sugen/hypoxia (SuHx)-induced PAH, SIN3a overexpression inhibits PASMCs proliferation and augments BMPR2 expression by inhibiting the methylation of its promoter region. RNA sequencing analysis reveals that SIN3a blocks the expression of the DNA methyltransferase DNMT1 and the histone methyltransferase enhancer of zeste homolog 2 (EZH2), reduces the activity of HDAC1, and decreases the enrichment of the EZH2-induced repressive marker H3K27me3 within the BMPR2 promoter region. Concurrently, SIN3a also enhances the level of DNA demethylation in the BMPR2 promoter region, mediated by DNA demethylase ten-eleven translocation 1 (TET1). Mechanistically, SIN3a promotes BMPR2 expression by reducing the binding of CCCTC-binding factor (CTCF, a key transcriptional suppressor of BMPR2) to the BMPR2 promoter. Intratracheal delivery of adeno-associated virus serotype 1 human SIN3a is a promising therapeutic approach in PAH by alleviating pulmonary vascular and right ventricular remodeling, decreasing right ventricular systolic pressure (RVSP) and mean pulmonary arterial pressure (mPAP), and reinstating BMPR2 expression (Fig. 3e).^289^ In summary, under SuHx-induced conditions, SIN3a exerts its influence on the progression of PAH by regulating epigenetic regulatory factors. This study holds significant therapeutic significance as it unveils a novel avenue for the use of SIN3a gene therapy to treat PAH by targeting the EZH2-H3K27me3-TET1 pathway in PASMCs.

#### Non-coding RNA regulation

In the 1950s, with the discovery of rRNA and tRNA, the crucial role of non-protein coding RNA molecules in gene expression was confirmed. Following the sequencing of the human genome in 2001, it was found that the majority of the human genome does not encode proteins. Instead, it highlighted the prominent role of non-coding RNA in the genome. More recently, non-coding RNAs, such as microRNAs (miRNAs) and long non-coding RNAs (lncRNAs), have received significant attention in the literature due to their regulatory roles in the target genome. Several studies have now linked post-transcriptional regulation by non-coding RNAs to cardiovascular diseases.

Currently, the interplay between miRNAs and chromatin in mammalian systems is less evident compared to lncRNAs. However, numerous studies have described their roles in gene regulation, and there is evidence supporting their involvement in the regulation of endothelial genes under conditions of hypoxia or disturbed blood flow.^290,291^ Generally, miRNAs regulate gene expression by inhibiting mRNA translation or promoting mRNA degradation.^292–294^ Dicer, a crucial endoribonuclease, is required for the biogenesis of miRNAs and siRNAs.^295^ In ECs, knockout of the Dicer gene leads to changes in the overall levels of miRNAs, resulting in alterations in the expression of endothelial-specific genes, including *eNOS*, and reduced formation of ECs networks in vitro.^290^ The changes in overall levels of miRNAs are associated with hypoxic conditions. In a hypoxic environment, the protein levels of Dicer decrease, resulting in reduced synthesis of miR-185. HIF-2α, as a direct target of miR-185, exhibits increased expression levels as miR-185 decreases. The upregulation of HIF-2α contributes to the maintenance of the induction of hypoxia-responsive genes in ECs under hypoxic conditions (Fig. 3f).^296^ miRNAs can also conditionally regulate eNOS. Under normoxic conditions, hnRNP E1 protects the eNOS 3’-UTR from recognition by miRNAs. However, under hypoxic conditions, an increase in serine phosphorylation of hnRNP E1 mediated by Akt and enhanced nuclear localization of hnRNP E1 disrupt the interaction between hnRNP E1 and the eNOS 3’-UTR, making the degradation of *eNOS* mRNA by miR-765 easier (Fig. 3f). These mechanisms greatly destabilize *eNOS* mRNA and decrease the overall expression of eNOS in ECs under hypoxic conditions.^297^

LncRNAs are transcripts of more than 200 nucleotide pairs that do not encode proteins. Instead, they play a role in the regulation of fundamental biological processes at both the transcriptional and post-transcriptional levels through epigenetic mechanisms.^298,299^ They can also influence gene expression by regulating processes such as transcription, splicing, nuclear export, or translation of mRNAs.^300^ Additionally, many lncRNAs can function as “sponges” for microRNAs, binding to microRNAs that play crucial roles in various human diseases. This binding helps alleviate the inhibitory effects of microRNAs on downstream target mRNAs.^301,302^ The first lncRNA described in ECs is sONE (also known as ATG9L2), which overlaps with the *NOS3* gene and regulates the expression of eNOS through post-transcriptional mechanisms. Importantly, under hypoxic conditions, sONE promotes the downregulation of *eNOS* mRNA in ECs.^220^ sONE is strongly induced and translocated to the cytoplasm under hypoxic conditions, where it can interact with *eNOS* mRNA and destabilize it (Fig. 3f). This discovery highlights for the first time the crucial role of lncRNAs in environmental adaptation, particularly in the context of hypoxic response, within vascular endothelium.^297^ In PAH, HPVR is associated with excessive proliferation of PASMCs.^303^ lncRNAs possess potent regulatory functions that can influence the proliferation, migration, and apoptosis of PASMCs. For instance, studies have shown that lncRNA-MEG3 can promote the proliferation and migration of PASMCs by activating downstream pathways of hypoxia signaling. Under hypoxic conditions, the expression level of lncRNA-MEG3 is significantly upregulated, which is achieved through a mechanism dependent on HIF-1α. Mechanistically, under hypoxic conditions, increased lncRNA-MEG3 directly interacts with miR-328-3p and leads to its degradation, subsequently promoting the expression of IGF1R, L-type calcium channel-α 1C (CaCNa1C), and PIM1. This ultimately contributes to pulmonary vascular remodeling and the development and progression of HPAH (Fig. 3g).^304^

Additionally, the expression of lncRNAs appears to be a sensitive indicator of cardiovascular diseases and can be used to differentiate the hemodynamic changes in ischemic and non-ischemic cardiomyopathy. Previous research has primarily focused on the gene expression profiles of protein-coding mRNAs to gain a better understanding of the molecular mechanisms underlying heart failure.^305^ However, these studies have recently expanded to include the field of lncRNAs, among others, offering possibilities for more comprehensive and in-depth research. Yang et al.^306^ compared myocardial tissues from normal donor hearts and those with ischemic and non-ischemic cardiomyopathy and found that the expression profiles of mRNAs, miRNAs, and lncRNAs could differentiate normal myocardium from ischemic and non-ischemic myocardium. However, only the expression profile of lncRNAs, and not mRNAs or miRNAs, could differentiate between ischemic and non-ischemic cardiomyopathy.

In sum, hypoxia exerts its influence on the occurrence and progression of cardiovascular diseases through a myriad of epigenetic modifications, including post-translational modifications of histones and non-histone proteins, DNA methylation, and non-coding RNA regulation. PTMs of histones encompass various facets of cardiovascular diseases, including the response of ECs to hypoxic signals in atherosclerosis and PAH. Recently, lactylation, an emerging modification, has commanded attention for its critical role in governing gene transcriptional regulation. Intriguingly, in the context of MI, lactylation stands implicated in myocardial fibrosis and the impairment of cardiac function. Furthermore, lactylation-mediated macrophage polarization assumes a pivotal role in the realm of inflammatory diseases. In the case of PAH, hypoxia-induced glycolytic shift and lactylation modification may facilitate HPVR. Furthermore, the demethylation of the BMPR2 promoter region significantly ameliorates pulmonary vascular and right ventricular remodeling, decreasing RVSP and mPAP. The intratracheal delivery of adeno-associated virus-encapsulated “beneficial genes” represents a promising therapeutic approach. Significantly, an intimate relationship exists between cardiovascular diseases and the intricate landscape of DNA methylation, alongside the post-transcriptional regulatory machinations orchestrated by non-coding RNAs.

### Natural rhythm in hypoxia signaling of cardiovascular diseases

#### Circadian rhythm

During the evolution of organisms in the natural environment under the long-term effect, the formation of many natural rhythms. From the earliest anaerobic conditions on earth to the oxygen-rich atmosphere facilitated by photosynthesis, changes in oxygen concentrations have accompanied and facilitated biological evolution. Especially since the emergence of advanced life, oxygen has become one of the basic life support. Thus, changes in oxygen concentration (including hypoxia) are also involved in the regulation of many biological natural rhythms, such as circadian rhythms and hibernation. Many living organisms adjust their behavior and physiology in response to Earth’s approximately 24-h rotation around the sun by relying on a biological clock that regulates rhythmic oscillations within their bodies, which is referred to as circadian rhythm.^307^ The molecular pathways responsible for regulating circadian rhythms is widely preserved and present in nearly all cell types.^308^ Prior research indicated that the transcriptional reaction to hypoxia varies among tissues and is influenced by the time of day, and the mRNA levels of *Hif1a* exhibit circadian oscillation in the cardiac tissue of mice.^85,309^ Additionally, experiments conducted in a controlled cell culture environment demonstrated that daily fluctuations in oxygen concentration, resembling those found in tissues, can effectively synchronize the internal clocks of cells that were initially unsynchronized. Of note, rhythmic variations in oxygen levels regulate the circadian rhythm via HIF-1α.^310^ It is important to consider the spatial and temporal differences in the response to hypoxia when studying both its harmful and beneficial effects.^311,312^

**Fig. 4:**
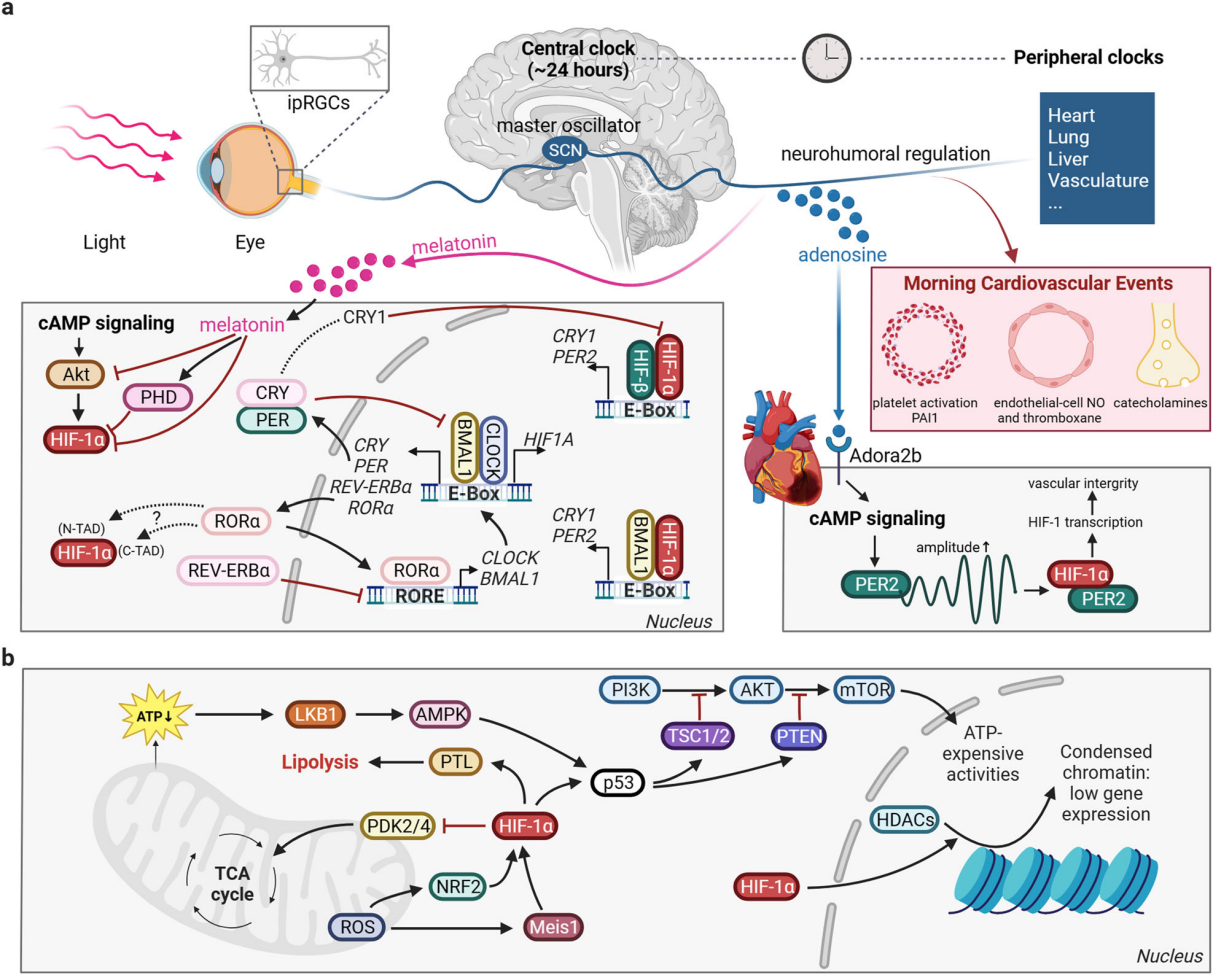
HIFs associated with natural rhythms in cardiovascular diseases. **a** HIFs related to circadian rhythms. **b** HIFs related to hibernation. (Created with *BioRender.com*). Abbreviations: Adora2b adenosine A2b receptor, AKT protein kinase B, ATP adenosine triphosphate, BMAL1 brain and muscle Arnt-like protein 1, cAMP cyclic adenosine monophosphate, CLOCK circadian locomotor output cycles kaput, CRY cryptochrome, E-BOX enhancer box, ipRGCs intrinsically photosensitive retinal ganglion cells, LKB1 liver kinase B1, Meis1 myeloid ecotropic viral integration site 1 homolog, mTOR mechanistic target of rapamycin, NRF2 nuclear factor erythroid 2-related factor 2, p53 tumor protein p53, PAI1 plasminogen activator inhibitor-1, PER period, PI3K phosphatidylinositol 3-kinase, PTEN phosphatase and tensin homolog, PTL protein triacylglycerol lipase, REV-ERBα reverse erythroblastosis virus α, RORE retinoic acid receptor-related orphan receptor response elemen, RORα retinoic acid receptor-related orphan receptor alpha, SCN suprachiasmatic nucleus, TSC1/2 tuberous sclerosis complex 1/2

In the pathological process of cardiovascular diseases, ischemia-reperfusion (I/R) injury is the most researched disease related to circadian rhythm (Fig. 4a).^313^ The I/R injury occurs when blood flow to an organ is obstructed followed by reperfusion, which leads to a heightened inflammatory response and collateral tissue damage upon reperfusion.^314,315^ During ischemia, the organ becomes profoundly hypoxic, and this leads to the stabilization of hypoxia-inducible transcription factors, enabling the ischemic tissues to adapt to limited oxygen availability by enhancing glycolytic capacity and attenuating hypoxia-driven inflammation.^313,316,317^ Mechanically, input from the hypothalamic master pacemaker centrally regulates the circadian mechanism, synchronizing the body’s cells to their intrinsic body time. In mammals, the suprachiasmatic nucleus (SCN) serves as the central clock located within the hypothalamus receiving light signals transmitted from photoreceptors in the eyes.^318^ It plays a vital role in synchronizing the daily rhythms of peripheral clocks found in various cells throughout the body (e.g., heart, and aorta).^308^ Furthermore, a feedback loop forms between HIF-1 and molecules involved in circadian rhythms (Fig. 4a). HIF-1 is capable of expressing *PER2* and *CRY1* genes,^83^ while HIF-1α can coexist with BMAL1 on E-box regions to enhance the expression of circadian and HIF target genes.^83,84^ Furthermore, the C-terminal helix of CRY1, known as the predicted coiled coil, modulates the duration and amplitude of oscillation. It directly interacts with HIFs, concealing their DNA-binding zone (bHLH domain), decreasing HIF’s stability and transactivation.^83,319^ Additionally, CLOCK and BMAL1 can induce the expression of the *HIF1A* gene,^84^ while PER:CRY complex suppresses CLOCK:BMAL1 dimers’ transactivation.^320^ In addition, BMAL1 and RORα cooperate to enhance the expression of target genes regulated by the N-TAD and C-TAD domains, however, without any indication of direct interaction with HIF-α.^321^

Moreover, it is widely acknowledged that the binding characteristics of circadian molecules (e.g., PER2, and CLOCK) and HIF-1α exhibit homology of the bHLH-PAS domain superfamily, enabling a crosstalk between the CLOCK and HIF-1α in the regulation of vasopressin gene expression in the SCN.^322^ This interaction may involve mediators of hypoxia-induced responses and overlapping properties of circadian pathways for target gene expression.^318^ Moreover, PER2, with a higher amplitude, serves as an influential molecule in facilitating the binding of HIF-1α to the promoter regions of its target genes downstream.^323^ Diurnal variations were observed in cardiac HIF-1α protein levels, transcript levels of its isoforms *Hif1.1* and *Hif1.2*, and the glycolytic enzymes *Pdk1* and *Ldh* over 24 h. And *Per2*^*−/−*^ mice exhibit a complete loss of circadian rhythmicity in HIF-1α after *Per2* deletion.^85^ *Per2*^*−/−*^ mice, which have notably larger myocardial infarct sizes, do not benefit from ischemic preconditioning, an experimental technique controlled by hypoxia signaling.^6,73,85^ Under normoxia, light-induced circadian overexpression of endothelial PER2 can simulate hypoxia response, inducing HIF-1α-mediated glycolysis while simultaneously regulating mitochondrial respiration or endothelial barrier function.^86^ These findings imply that strategies aimed at enhancing the amplitude of PER2 could precondition the myocardium by creating a signaling environment similar to HIF-1 prior to an ischemic event. Furthermore, chronic hypoxia disrupts the circadian rhythm in mice, causing arrhythmia under constant darkness. The underlying reasons could be attributed to a partial decoupling of central and peripheral clocks due to impaired adaptation to chronic hypoxia, similar to the effects of restricted feeding.^324^ Arrhythmicity during periods of increased oxygen consumption, such as activity and food intake, may occur in mice during their dark phase.^310^

Clock-enhancing strategies hold promise as potential treatments for various diseases, including I/R injury, due to the observed correlation between circadian amplitude dampening and disease progression.^325^ Light, however, is a complex entity characterized by its photoperiod (duration), illuminance (intensity), and wavelength. Short-term intense light exposure (10,000 lx) has been shown to provide endothelium-dependent cardioprotection in humans (for 30 min every morning for 5 days) and in mice (for 3, 5, or 7 consecutive days with a *14* *h (hours):10* *h* *L(light):D(dark) cycle*). This effect is achieved through the interaction of augmented PER2 amplitude and HIF-1.^86^ Furthermore, a 24-h photoperiod of high-intensity (1400 lx) blue spectrum light with a peak wavelength of 442 nm prior to I/R considerably mitigates organ damage severity.^87^ When mice were subjected to a 24-h period of blue spectrum light exposure, there was a noticeable decrease in their sympathetic tone, as indicated by lowered heart rate and low frequency (LF) / high frequency (HF) ratio (LF/HF ratio stands for heart rate variability (HRV)). While acute exposure to white or fluorescent light can increase sympathetic activity, prolonged exposure, especially to blue spectrum light, may lead to a decrease in adrenergic tone. This holds significant potential, especially in the realm of perioperative medicine, where patients scheduled for elective surgery could receive preoperative circadian therapeutics as a means to mitigate the effects of I/R injury. Studies found a diurnal pattern in troponin values after aortic valve replacement. Troponin values were higher in the morning post-surgery compared to the afternoon, while afternoon surgery correlated with lower incidences of major adverse cardiac events.^326^ Thus, applying pre-surgical light therapy to enhance circadian amplitude may provide robust cardioprotection for high-risk non-cardiac or cardiac surgery.

Collectively, modifying the light spectrum may offer therapeutic utility in sterile forms of cellular injury, akin to the protective effects of ischemic preconditioning, which will be discussed further in the subsequent sections on the interplay between different organs/systems (See the Section "Remote regulation of hypoxia signaling in cardiovascular diseases"). But on the contrary, if excessive hypoxia will break the balance of circadian rhythm. In particular, hypoxia has the potential to cause metabolic adaptations to an acidic microenvironment, which has been observed to be capable of inducing circadian disruptions.^320^ The circadian disruption could be reversed by buffering the media to a neutral pH in hypoxic conditions. The change in pH observed in hypoxia was influenced, at least in part, by HIFs, as the simultaneous knockdown of HIF-1/2α resulted in a restoration of pH towards neutral and a partial restoration of circadian rhythmicity.^327^ (See the Section "Remote regulation of hypoxia signaling in cardiovascular diseases").

#### Hibernation

In addition to the circadian rhythm, another biological rhythm commonly found in nature related to sleep is hibernation. Under such circumstances, it is of great clinical significance to explore how organisms maintain the basic operation and steady state of the body with low energy consumption in the form of "big sleep" to resist harsh environments (such as low temperature, limited nutrient availability, a period of low oxygen supply, or even, long-term interstellar voyages), and the occurrence of complications (such as pressure sores, thrombosis, etc.). Hibernating (quiescent and torpid) cells exhibit a metabolic transition from glucose-driven OXPHOS to FAO.^328^ The HIF-1α signaling pathway exhibits significant activation during the hibernation state of various animals, such as 13-lined squirrels, little brown bats (*Myotis lucifugus*), as well as B6N and B6J mice.^329–331^ As Fig. 2a shows, HIF-1α facilitates FAO through upregulation of PDK2/4 and subsequent downregulation of pyruvate dehydrogenase (PDH), which inhibits the first step of the TCA cycle, and enhances resistance against hypoxia stress and oxidative damage (Fig. 4b).^6^ During the process, inhibiting PDH reduces carbohydrate utilization by limiting the flow of glycolysis products to the TCA cycle and promoting the β-oxidation of ketones and fatty acids. Hematopoietic stem cells (HSCs) in the bone marrow reside in a hypoxic niche. To maintain their quiescent state, HSCs rely on glycolysis and β-oxidation, regulated by HIF-1α, for low levels of ATP generation.^332^ Simultaneously, the protein triacylglycerol lipase (PTL), as another transcriptional targets of HIF-1α, is responsible for promoting lipolysis by degrading adiposomes (fat droplets), releasing fatty acids to facilitate FAO.^328^ PTL exhibits significant lipolytic activity at low ambient temperatures, which is an inherent characteristic of the protein in all mammalian lineages. PTL has been observed upregulated in HSCs to ensure the supply of fatty acids,^333^ and PTL upregulation is a distinctive characteristic exclusively observed in hibernating animals.^334^ Furthermore, hibernating ground squirrels exhibited increased expression of PDK4 and PTL in the white adipose tissue, heart, and skeletal muscle.^335–337^ In addition, HIF-1α expression in HSCs is regulated by the DNA-binding transcription factor known as myeloid ecotropic viral integration site 1 homolog (Meis1).^338^ The well-established role of Meis1 in counteracting oxidative stress involves serving as an upstream regulator that activates HIF-1α in response to elevated levels of intracellular ROS and low oxygen levels.^338–341^ Moreover, mitochondrial ROS production elevation additionally triggers HIF-1α by means of the oxidative stress-responsive nuclear factor erythroid 2-related factor 2 (NRF2), thereby hindering mitochondrial respiration and subsequently activating liver kinase B1 (LKB1)/AMP-activated protein kinase (AMPK).^338,342,343^ However, under such ATP-deprived conditions caused by nutrient scarcity or hypoxia, the energy-sensing LKB is activated, along with the downstream AMPK, preceding the upregulation of HIF-1α and triggering autophagy.^328,344^ Furthermore, the potential activity of p53 released by sustained HIF-1α accumulation may trigger the activation of PTEN and TSC1/2, which inhibits energy-intensive PI3K/AKT/mTOR pathway and is essential to maintain quiescence and topor.^6,328,345^ In addition to creating an antioxidant environment, HIF-1α also enlists HDACs to reduce histone acetylation and compact DNA into heterochromatin, leading to enhanced radiation resistance.^346^ Besides HIF-1α, hibernators rely on the hypoxia-related RBM3 to safeguard their cells through sustaining protein equilibrium amidst low metabolic circumstances.^347^

During hibernation, animals experience significant physiological, morphological, and behavioral transformations. For instance, the body temperature of seasonal hibernators in cold environments dramatically drops to a range of 0–4 °C, while heart rate and respiration decrease by 95%. Additionally, there is a notable reduction in renal function.^348^ While the precise mechanistic regulation of the transition from glucose to fat metabolism during hibernation remains incompletely understood, it appears to share similarities with the mechanisms observed in instances of starvation, diabetes, and caloric restriction.^349–352^ And HIF-1α expression during hibernation may vary across tissues and is potentially linked to species-specific differences in gene expression patterns, which needs deeper investigations. Therefore, investigating the specific role of HIFs in hibernation in order to seek clinical translation in warm-blooded mammals, such as inducing a low-metabolic "hibernation" state in relevant organs, tissues, and cells during acute cardiovascular events for rejuvenation, repair, and therapeutic opportunities, would hold significant implications.

### Intracellular interactions and remote regulations of hypoxia signaling in cardiovascular diseases

With the introduction of the concept of panvascular medicine, the significance of hypoxia on the progress and treatment of cardiovascular diseases will need to take into account the overall aspects of the body. This involves the impact of systemic hypoxia on the body and the interactive impact of a local (tissue and organ) hypoxia on others (tissues and organs). This hypoxia-induced organ/tissue interaction is mainly through the neurohumoral pathway, that is, often after a certain organ or tissue is stimulated (such as ischemia and hypoxia), release the corresponding secretory factors to the surrounding environment and then circulate through the body fluid to the target organ to simulate the stimulation and construct the corresponding effects, which is generally slower. A faster response is local stimulation that excites the receptors and afferent nerves at the corresponding sites, acting on the effector organ/tissue through the reflex arc. The two approaches can (and in most cases do) complement each other.

#### Cardiovascular control of central circadian clock

Cardiovascular pathology and thrombotic formation possess an inflammatory procedure and are subject to circadian influences on both local and systemic scales.^353^ Furthermore, in response to a diminishing photoperiod, energy reserves are directed towards internal mechanisms, such as immune function, that are crucial for adapting to seasonal survival strategies.^87^ Physiological rhythms that contribute to morning cardiovascular events involve platelet activation, production of endothelial-cell NO and thromboxane,^354^ increased production of prothrombotic plasminogen activator inhibitor 1,^355^ and elevated catecholamine levels.^356^ The peak occurrence of intrinsic electrical conduction and arrhythmogenic abnormalities is also observed during the early daytime.^357,358^ Nocturnal blood pressure dipping, an abnormal phenomenon, serves as an independent prognostic indicator of cardiovascular risk, regardless of daytime blood pressure levels.^359^ Furthermore, epidemiological evidence reveals a distinct rise in the occurrence of myocardial infarction^360,361^ and aortic rupture^362^ during the morning hours and during transitions associated with daylight saving time. Or rather, a rise in myocardial infarctions have been observed across all U.S. states during the darker winter months.^363^ Moreover, long-term night shift work linked to increased myocardial infarct size in patients with myocardial infarction.^364^ The exact mechanism by which the entrainment signal related to central clock reaches peripheral organs remains uncertain, but it could involve neuro-hormonal factors or autonomic innervation.^307^ However, specific phototherapy treatments can actually provide protective effects in simulating cardiac hypoxia/ischemia preconditioning as mentioned above. In particular for interaction of hormonal factors as a remote regulator, adenosine released into circulation, regulated by central lock, can bind to Adora2b and subsequently activated cardioprotection patterns.^85,86^ A_2B_ adenosine receptor (ADORA2B) signaling has been demonstrated to deneddylate CUL1 and inhibit proteasome activity for PER2, while simultaneously enhancing cAMP signaling to stablize PER2 transcription. Consequently, this amplifies PER2’s amplitude and facilitates its interaction with HIF-1, potentially improving oxygen efficiency for cardioprotection (Fig. 4a). ADORA2B signaling has been identified as a key aspect of HIF-dependent cardioprotection. Thus, purinergic receptor agonists, including the ADORA2B, have been identified as promising therapeutic candidates for the treatment of I/R injury.^85,365^ Moreover, melatonin, also referred to as N-acetyl-5-methopxytryptamine, is a hormone produced by the pineal gland (regulated by SCN) in the brain to regulate the circadian rhythm. The potent HIF-1α inhibition activity of melatonin and its derivatives, including N-butyryl-5-methoxytryptamine (NB-5-MT), has been demonstrated in recent studies, employing multiple molecular mechanisms (Fig. 4a).^366^ Melatonin and NB-5-MT activate PHDs, promoting HIF-1α degradation through interaction with VHL.^367–369^ Melatonin increases VHL activity by suppressing Akt/GSK-3β, reducing HIF-1α stability.^370^ Additionally, melatonin inhibits HIF-1α expression by impairing its translation.^369,371^ The CLOCK-HIF-1α signaling pathway and the melatonin-superoxide dismutase (SOD)-catalase (CAT) signaling pathway (involving SIRT1) have been proposed to play contrasting roles in inflammation, with the former promoting during the acute phase and the latter inhibiting during the quiescent phase.^372^ This suggests the existence of a "switch" within these pathways. However, the precise mechanisms in the cardiovascular system remain unclear.

In addition, besides the vascular impact caused by compromised rhythmic regulation, disruptions in clock function within adipose tissue, liver, and muscle can contribute to the development of cardiometabolic disorders.^353,373^ Clock function in muscles plays a crucial role in regulating glucose uptake and exercise capacity, which can have long-term implications for cardiovascular risk. Several ratelimiting enzymes involved in cholesterol and bile acid metabolism exhibit diurnal variations within the liver. When these internal cycles become misaligned with food intake, it can potentially contribute to dyslipidemia. Furthermore, the misalignment of feeding patterns with circadian cycles, which regulate adipose insulin sensitivity, nutrient retention, inflammatory response, and thermogenesis, can potentially contribute to metabolic complications associated with obesity.

Despite our understanding of the profound impact of the circadian clock on human health, the clinical application of this knowledge is hindered by the challenge of detecting and diagnosing circadian disruption, as well as the need to effectively translate insights from cellular and tissue-level circadian clock research into clinical practice. Given this situation, currently, the application of circadian rhythms in cardiovascular medicine is mainly limited to the integration of organ metabolism and drug application associated with circadian rhythm. The rhythmic expression of key mitochondrial enzymes in the liver, which play a vital role in activating or metabolizing lipid-soluble drugs, can affect the pharmacokinetics at different times of the day.^374^ Correspondingly, drug targets themselves may exhibit peak activity at distinct periods. For example, the rate-limiting enzyme of cholesterol biosynthesis is HMG-CoA reductase, which peaks at night in humans. This observation prompted the recommendation to administer short-acting statins in the evening. Agents with a half-life of less than 12 h exhibit optimal efficacy when their delivery is aligned with the inherent circadian rhythm.

#### Intercellular lactate shuttle

The lactate shuttle theory primarily explains the intracellular and intercellular movement of lactate within and between cells, summarizing the complete process of lactate migration across cell membranes.^375,376^ The intercellular lactate shuttle is a metabolic phenomenon where lactate, a byproduct of glucose metabolism, is transported between cells and tissues to be utilized as an energy source.^375,377^ It involves the conversion of glucose to lactate in active muscle cells during intense exercise or periods of high energy demand. The lactate is then released into the bloodstream and taken up by other tissues, such as the heart, liver, and brain, where it is converted back to glucose or used as a fuel for energy production. This dynamic process allows lactate to serve as a vital energy substrate and plays a crucial role in maintaining energy balance and optimizing overall metabolic efficiency.

**Fig. 5:**
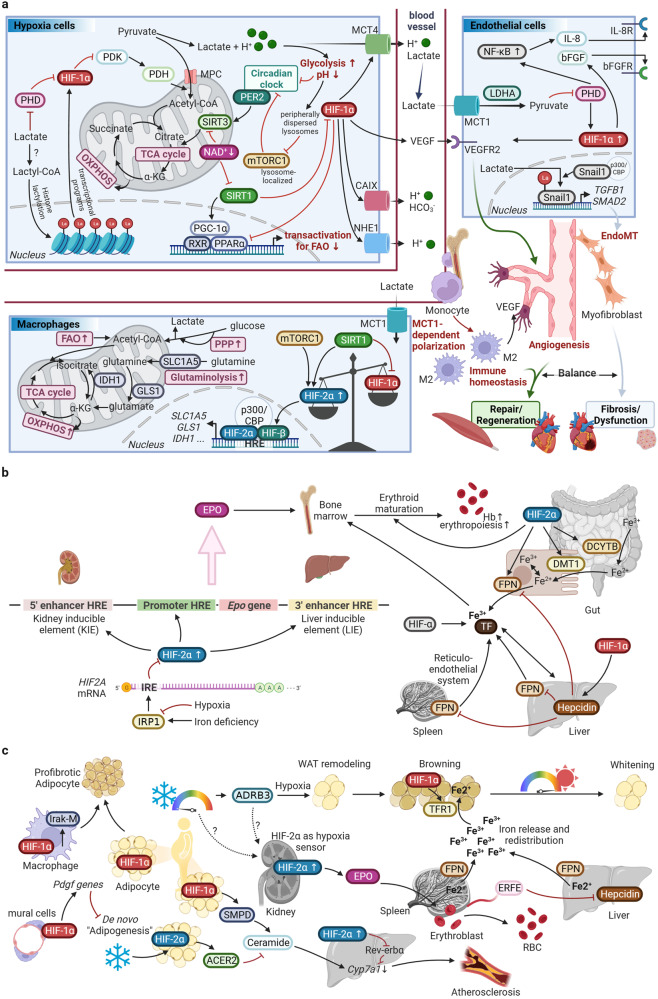
Intracellular interactions and remote regulations of hypoxia signaling in cardiovascular diseases. **a** HIF-αs in intercellular lactate shuttle. **b** HIF-αs in EPO generation. **c** Relationship between HIF-αs in cardiovascular diseases and adipose tissue. (Created with *BioRender.com*). Abbreviations: ACER2 alkaline ceramidase 2, ADRB3 β3-Adrenergic receptors, bFGF basic fibroblast growth factor, bFGFR basic fibroblast growth factor receptor, CAIX carbonic anhydrase IX, DCYTB duodenal cytochrome b reductase 1, DMT1 divalent metal transporter 1, ERFE erythroferrone, FPN ferroportin, GLS1 Glutaminase 1, IDH1 Isocitrate Dehydrogenase 1, IL-8 Interleukin-8, IL-8R Interleukin-8 Receptor, Irak-M interleukin-1 receptor-associated kinase M, IRE iron-responsive element, IRP1 iron-regulatory protein 1, M2 M2 macrophage, MPC mitochondrial pyruvate carrier, mTORC1 mechanistic target of rapamycin complex 1, NAD+ nicotinamide adenine dinucleotide, NF-κB nuclear factor-kappa B, NHE1 sodium-hydrogen exchanger 1, Pdgf platelet-derived growth factor, PGC-1α peroxisome proliferator-activated receptor gamma coactivator-1 alpha, pH potential of hydrogen, RBC red blood cell, RXR retinoid × receptor, SLC1A5 Solute Carrier Family 1 Member 5, SMPD sphingomyelin phosphodiesterase, TF transferrin, TFR1 transferrin receptor 1, VEGFR2 vascular endothelial growth factor receptor 2, WAT white adipose tissue

HIFs induce an acidic microenvironment both inside and outside cells, which serves as the foundation for lactate diffusion in the body. To sustain sufficient energy levels within cells when exposed to hypoxic conditions, there is a preference for glycolysis, which results in enhanced utilization of glucose and the generation of lactate (Fig. 5a).^378^ Under hypoxia, the increased expression of PDK1 results in the inhibition of the enzymatic function of PDH. As a consequence, the conversion of pyruvate to acetyl-CoA, which is necessary for its entry into the TCA cycle, is impeded. Instead, there is a preference for the production of lactate.^1,6^ Furthermore, cells can regulate their intracellular pH levels by upregulating the activation and expression of certain HIF-1-dependent genes.^379^ These genes include *SLC16A3*, encoding monocarboxylate transporter 4 (MCT4), which is predominantly found in highly glycolytic cells and helps eliminate lactate and hydrogen ions.^380,381^ MCT4 and mitochondrial pyruvate carrier (MPC) are supposed to mediate metabolic rewiring of cardiomyocytes, featuring a decrease in mitochondrial pyruvate oxidation and an increased export of lactate, thereby exacerbating heart failure.^382,383^ MPC serves as the gateway for the glycolytic final product pyruvate to enter the mitochondria. It has been proposed to reestablish a balanced pyruvate-lactate axis to relieve hypertrophy/heart failure by suppressing the lactate outflow through MCT4 and maintaining MPC-mediated mitochondrial inflow of lactate.^382^ Of note, the reduction of MCT4 was observed to inhibit multiple HIF-1 transcriptional activity in a lactate-independent approach in gliboblastoma.^384^

Another gene involved is *CA9*, encoding carbonic anhydrase IX (CAIX), which aids in the production of bicarbonate and hydrogen ions from carbon dioxide and water.^385^ Moreover, HIF-1α, induced by low pH and hypoxia, can initiate the activation of sodium-hydrogen exchanger 1 (NHE1). This exchanger facilitates the exchange of intracellular hydrogen ions with extracellular sodium ions.^6,379^ Moreover, abnormal mTORC1 signaling is responsible for mediating the disruption of the circadian clock caused by hypoxia-induced acidification.^327^ Acid, a byproduct of HIFs-directed metabolism, redistributes lysosomes to the cell periphery (away from its normally activated and perinuclear position, spatially separated from its upstream RHEB), which disrupts mTORC1 signaling and clock protein translation.^327^ Inhibiting acidification, restoring mTORC1, or targeting HIF-1α*/*LDHA axis rescues clock oscillation, revealing acid-mediated inhibition of mTORC1 halting the clock.^327^ Furthermore, the significant entraining influence (zeitgeber) for peripheral clocks is now recognized to be feeding cycles.^386^ Consequently, it is particularly captivating to contemplate the possibility that mTOR, with its ability to perceive nutrients, growth factors, and energy levels and coordinate cellular response, may play a crucial role in this synchronization pathway, which is currently not well comprehended.

Endothelial cells (ECs), which line the inner walls of blood vessels, serve as gatekeepers of metabolism by regulating oxygen and nutrient delivery to tissues through angiogenesis, while predominantly relying on glycolysis to generate energy by metabolizing glucose into lactate.^387–391^ In vitro, lactate enhances the responsiveness of ECs to VEGF by increasing the content of VEGFR2 through enhanced stabilization of HIF-1α, while the accompanied activation of an autocrine HIF-1/bFGF and NF-κB/IL-8 signalings further proof lactate-induced angiogenesis.^392–394^ In vivo, ECs utilize glycolysis for lactate shuttling to drive muscle regeneration from ischemia (Fig. 5a). The loss of *pfkfb3* a key glycolytic regulator in ECs, impairs muscle revascularization and regeneration. EC-derived lactate instructs macrophages to adopt a proangiogenic and proregenerative phenotype. Increasing lactate levels during ischemia improves M2-like macrophage polarization in an MCT1-dependent manner and enhances muscle reperfusion and regeneration.^387^ Furthermore, in M2 macrophages, upregulated HIF-2α levels have been observed, while M1-polarized macrophages are reported to exhibit induced HIF-1α expression.^149^ PHD2 haplodeficiency in mice was shown to shift macrophages to an M2 phenotype without affecting HIF-1α levels. This resulted in a pro-arteriogenic phenotype, effectively preventing hind-limb ischemia in a murine model.^395,396^ Furthermore, M2 macrophages display elevated arginase 1 levels, regulated by HIF-2α, which competes with inducible nitric oxide for the shared substrate L-arginine.^149,397^ Activated M2 macrophages exhibit elevated HIF-2α expression and are characterized by enhanced FAO, pentose phosphate pathway (PPP) and mitochondrial respiratory chain activity.^398^ Additionally, sustained acidosis—activated SIRT1 could deacetylate HIF-1/2α, which inhibits HIF-1α but activates HIF-2α promoting glutamine anaplerosis and reductive carboxylation, to fully replenish intermediate products and mobilize the TCA cycle, especially in cancer cells.^399^ The SIRT1/HIF-2α axis enhances the expression of SLC1A5 (increasing glutamine import into mitochondria), GLS1 (converting glutamine to glutamate), and IDH1 (redirecting α-ketoglutarate towards isocitrate). Glutamine deprivation exerts a negative impact on M2-polarized macrophages while leaving M1 macrophages unaffected.^400^

However, excessive pro-angiogenic molecules can impair EC function, preventing normal angiogenesis and disrupting the balance between inflammation and blood vessel formation. Lower vascular density, a high-risk factor for hypoxia and inflammation, is associated with diseases like cardiovascular diseases, obesity, and cancer.^401,402^ Furthermore, cardiac hypoxia after MI induces endothelial-to-mesenchymal transition (EndoMT), which plays a role in changing the endothelial phenotype towards mesenchymal cells like myofibroblasts. This process contributes to cardiac fibrosis and ultimately leads to cardiac dysfunction.^256,403,404^ Cardiac fibrosis, a pathological process of matrix remodeling and fibroblast activation, is unavoidable in cardiac injuries like MI. While initially preventing tissue rupture during MI, persistent cardiac fibrosis often leads to a poor prognosis and eventual heart failure.^253^ The Snail1-transforming growth factor-β(TGF-β)-Smad2/3 pathway can govern EndoMT-related fibrosis in various cardiovascular diseases.^257,258,405,406^ Later, it was discovered that elevated lactate levels significantly elevate cardiac fibrosis and worsen cardiac dysfunction through the induction of EndoMT in the myocardium after MI.^259^ Mechanistically, lactate was shown to introduce a relationship between CBP/p300 (crucial writers of histone lactylation^218^) and Snail1, promote the nuclear translocation and subsequent lactylation of Snail1 and thus enhance Snail1-TGF-β-Smad2 pathway following hypoxia/MI (Fig. 5a). Moreover, Snail is a direct target of HIF-1α by demonstrating that HIF-1α directly attaches to its HIF-1-binding regions within the *SNAIL* promoter in cultured human coronary endothelial cells, promoting their EndoMT.^407^ Furthermore, acute events typically characterize myocardial infarctions, whereas, for compensatory events like myocardial hypertrophy, the production of lactate by LDHA facilitates myocardial hypertrophy.^408^ In the context of end-stage cardiac events, specifically heart failure, the level of lactate in the serum is closely associated with the prognosis and mortality of heart failure patients or post-heart transplant recipients.^255,409,410^ Furthermore, lactic acid was found to induce myofibroblast differentiation where HIF-2α in lung vascular endothelial cells could pathogenically upregulate SNAIL1/2 and subsequent EndoMT, contributing to vascular remodeling, obliterative pulmonary vascular lesions, pulmonary fibrosis and advancement of severe pulmonary hypertension.^411,412^ In addition, EndoMT also plays a vital role in the development of various cardiovascular conditions, including myocardial infarction, atherosclerosis, cardiac fibrosis, and valvular diseases.^413^

Collectively, in the process of heart or tissue damage repair, induced by lactate shuttle, the damaged area undergoes the formation of new thin-walled capillaries, fibroblasts, and inflammation infiltration. This state bears some resemblance to the "granulation tissue" formed during repair of skin or epithelial tissue damage. The involvement of HIFs plays a role in its formation and outcome. The key factors determining whether the direction is towards regenerative repair or fibrosis and dysfunction are still subject to further exploration. However, early intervention appears to be particularly crucial based on existing evidence. In particular, timely initiation of reparative signals in monocyte-macrophages is crucial for restoring immune homeostasis and initiating the post-MI repair process.^414,415^ Early remote activation of reparative signals in bone marrow and peripheral monocytes post-MI shifts the monocyte response to an optimal state of healing, establishing a balance. Monocytes with metabolic reprogramming during early MI, promotes histone lactylation and activates repair-related gene expression.^415^ Histone lactylation acts as a crucial switch, regulating the dual activities of monocytes (anti-inflammatory and proangiogenic), promoting cardiac repair post-MI.^218^ The IL-1β-dependent GCN5 recruitment catalytically affects H3K18la, potentially regulating monocyte histone lactylation and downstream reparative gene expression after MI.^415^ Furthermore, MCT1 serves as the initiation point for the uptake of lactate by cells and the processes mentioned above. According to the Human Protein Atlas, MCT1 is present in endothelial cells, cardiac myocytes, and fibroblasts. While cardiac myocytes exhibit the highest level of MCT1, endothelial cells show twice the expression level compared to fibroblasts.^416^ Hence, cells with a higher abundance of MCT1 are more likely to take up lactate from the circulation. Considering the expression abundance of MCT1, the heart is primarily inclined towards self-repair (for myocardium) and complementary angiogenesis (for endothelial cells). During the later stages of disease progression, Snail-TGF-β-induced EndoMT emerges as a dominant factor. This is supported by findings from the Human Protein Atlas, which reveal that the expression levels of Snail1 in cardiac endothelial cells are notably higher compared to other cardiac cell types such as cardiac fibroblasts and cardiomyocytes.^416^ The prevalence of Snail overexpression in cardiac endothelial cells is closely associated with the development of organ fibrosis and functional impairment.

#### Interactions between EPO and cardiovascular systems

The systemic interplay of the hypoxia pathway in the body can be ascribed to diverse tissue constituents existing within the cardiovascular system, including not only adipose tissue (e.g., obesity, and perivascular fat) but also blood (e.g., hemoglobin). Alternatively, it may stem from the gradual deterioration of multiple organs within the cardiovascular system due to systemic aging. EPO was initially identified as being associated with HIF-1, playing a crucial role in the synthesis of RBCs and hemoglobin throughout the body. However, recent research suggests that HIF-2 appears to have an equally or even more significant impact on EPO synthesis.^417^ Given that RBCs serve as oxygen-carrying cells in the bloodstream, any alterations in their function and composition inevitably affect the cardiovascular system. HIF-1α is currently considered to enhance the transcription of proteins that reduce oxygen consumption and boost angiogenesis, while HIF-2α acts as the main trigger for the production of EPO, a hormone involved in RBC synthesis.^43,417,418^ From the perspective of multi-organ coordination, HIF-2α regulates EPO synthesis alongside iron homeostasis (Fig. 5b). HIF-2 boosts EPO synthesis in the kidney and liver, elevating its serum levels and stimulating bone marrow erythropoiesis.^417^ Hypoxia induces EPO in the liver via the liver-inducibility element (LIE) at the gene’s 3'-end. In contrast, renal EPO induction requires the kidney inducibility element (KIE) upstream of the transcription start site.^419,420^ LIE or KIE activation is crucial for HIF-2 to bind HRE promoter and induce EPO expression.^421,422^ In liver, HIF-2, while the isolated HRE exhibits a preference for binding HIF-1α, the HRE within the native *Epo* 3' enhancer shows a stronger affinity for HIF-2α.^423^ This preference is facilitated by cooperative interactions with other transcription factors that are bound to the promoter, the 3' enhancer, or other cis-acting sequences. A hepatic nuclear factor-4 (HNF-4) binding site within the enhancer may play a role in this process. However, the role of a putative and highly conserved 5'-HRE within the kidney-inducibility region remains unclear in vivo. Furthermore, a conserved iron-response element (IRE) is situated within the 5'-untranslated region (UTR) of *HIF2A* mRNA, enabling the translation of HIF-2α protein in response to elevated iron levels.^424^ Iron regulatory protein 1 (IRP1) serves as a key regulator in cellular iron metabolism. Reduced binding between IRP1 and the iron-responsive element (IRE) on *HIF2A* mRNA, particularly in conditions of hypoxia and sufficient iron supply, leads to elevated levels of HIF-2α protein expression and subsequent raised erythropoiesis and intestinal iron absorption.^425,426^ However, iron overload may lead to PHDs-induced HIF-α protein degradation. By contrast, iron deficiency may cause IRP1/IRE binding and translational repression of *HIF2A* mRNA, while PHDs-induced HIF-α protein degradation will also be inhibited. In the duodenum, duodenal cytochrome b reductase 1 (DCYTB) facilitates the conversion of Fe^3+^ to Fe^2+^, allowing the latter to be absorbed by enterocytes through divalent metal transporter-1 (DMT1). HIF-2 regulates both *DCYTB* and *DMT1*.^427,428^ Iron is subsequently released into the bloodstream through ferroportin (FPN), which is also induced by HIF-2.^429^ Ferroportin is the sole recognized cellular iron exporter, degraded by hepcidin. Iron is transported in the bloodstream as a complex with HIF-regulated transferrin, reaching various organs such as the liver, bone marrow, and others. HIF-regulated iron factors include transferrin (transports Fe^3+^ in plasma),^430–432^ TFR1 (transferrin receptor), ceruloplasmin (oxidizes Fe^2+^ to Fe^3+^ for transport),^433^ and haeme-oxygenase-1 (recycles iron from erythrocytes)^434^. Within the reticuloendothelial system, iron is acquired by cells through the process of phagocytosis, specifically targeting aging or senescent RBCs. Elevated erythropoietic activity in the bone marrow leads to the generation of GDF15 and erythroferrone, both of which inhibit hepcidin production in hepatocytes.^435^ Hepcidin (mainly derived from liver) suppression promotes increased FPN expression in enterocytes, hepatocytes, and macrophages, thereby enhancing iron absorption and utilization from internal reserves. Hepcidin (encoded by *Hamp1*) is directly regulated by HIF-1 through its binding to the *Hamp1* promoter.^436^ Furthermore, endothelial HIF-2 plays a specific role in erythroid maturation, involving vascular adhesion molecule (VCAM)-1, a surface protein supporting erythroid maturation.^437^ Furthermore, in *Irp1*-deficient mice suffering from pulmonary hypertension, the administration of MK-6482 to suppress HIF-2α has shown potential in restoring EPO levels back to normal range, reversing cardiac fibrosis, and preventing cardiomyocyte degeneration, thereby mitigating pulmonary hypertension.^438^ From a holistic perspective, maintaining the activation of the HIF signaling pathway within a normal range has been demonstrated to mildly enhance the expression of EPO and VEGF. This, in turn, contributes to improved metabolic and cardiovascular traits such as enhanced glucose tolerance, decreased total cholesterol and blood pressure levels, reduced presence of toxic metabolites, and lower inflammatory burden. Additionally, it helps prevent microvascular rarefaction and stimulates geroprotective vascular rejuvenation.^439,440^

#### Interactions between adipose tissue and cardiovascular diseases

Energy-storing white adipose tissue (WAT) can expand as energy storage demands increase. "Metabolically healthy" obesity shows healthy WAT expansion, favoring protective subcutaneous WAT depot expansion through adipocyte hyperplasia. Pathological WAT expansion involves limited expandability of beneficial subcutaneous WAT, leading to adverse tissue remodeling with adipocyte hypertrophy, chronic inflammation, and fibrosis.^441–443^ Obesity is associated with excessively expanded adipocytes, the growth and remodeling of adipose tissue, and the accumulation of lipids. Due to the restricted diffusion of oxygen into hypertrophic adipocytes, cellular hypoxia is evident within adipose tissue, which induces HIF-1α (Fig. 5c). These factors contribute to not only localized tissue hypoxia, but also dysfunction in adipose tissue.^444–447^ Significantly, adipose tissue serves as not only an energy storage organ but also an endocrine organ, releasing adipocytokines that impact cellular and tissue function across the entire body.^448^ Adipocyte HIF-2α confers a protective effect against insulin resistance induced by a high-fat diet (HFD), whereas adipocyte HIF-1α exacerbates HFD-induced obesity, insulin resistance, inflammation, and fibrosis within adipose tissue.^449–451^ PRDM16 induces FAO and ketogenesis in adipose cells, leading to the secretion of β-hydroxybutyrate (BHB). BHB blocks myofibroblast differentiation induced by HIF-1α or TGF-β and promotes beige adipocyte differentiation in precursor cells. This action of BHB depends on the ketyolytic enzyme BDH1, emphasizing the role of ketone metabolism in adipose tissue remodeling.^452^ Furthermore, elevated HIF-1α levels in mouse macrophages correlate with compromised glucose metabolism, adipose tissue fibrosis, inflammation, and heightened macrophage infiltration.^453^ HIF-1α in macrophages is believed to have a unique impact on adipose tissue dysfunction associated with obesity by targeting its downstream interleukin-1 receptor-associated kinase M (Irak-M).^453,454^ As Irak-M inhibits the toll-like receptor (TLR) pathway, its upregulation via HIF-1α is likely to suppress the pro-inflammatory response.^454^ Furthermore, emerging adipocytes in gonadal WAT derive from PDGFRβ+ perivascular cells, which closely resemble mural cells.^455^ An identified signaling pathway involving mural cells, which exhibit anti-adipogenic and pro-fibrogenic properties, relies on the HIF-1α-dependent PDGFR-ERK cascade. This cascade effectively suppresses the activity of PPARγ in PDGFRβ+ cells through the phosphorylation of PPARγ serine 112 (S112), acting in an autocrine/paracrine manner. As a result, this mechanism limits the occurrence of healthy de novo adipogenesis in obesity.^441^

In the context of atherosclerosis, aside from the localized vascular lesion itself, the interplay among various organs exerts a significant influence.^456^ Cold exposure has been found to suppress HFD-induced obesity, insulin resistance, adipose dysfunction, and dyslipidemia by enhancing adipocyte thermogenesis.^457,458^ Adipocyte HIF-2α increases with mild cold exposure (16 °C), which activates ceramide catabolism by targeting *Acer2* gene (encoding alkaline ceramidase 2), leading to improved atherosclerosis through cold-induced thermogenesis elevation and hepatic cholesterol elimination (Fig. 5c).^92^ Deficiency of HIF-2α in adipocytes impaired thermogenesis, leading to increased inflammation in adipose tissue and a deterioration in insulin sensitivity.^459^ Meanwhile, adipocyte-HIF-1α enhances ceramide levels by activating *Smpd3*. This, in turn, exacerbates atherosclerosis by inhibiting cholesterol elimination and amplifying both local and systemic inflammation levels. Overexpression of SMPD3 lentivirus and administration of ceramide counteract the benefits of adipocyte HIF-1α deficiency in atherosclerosis.^460^ Type 2-neutral sphingomyelinase (nSMase2), also known as SMPD3, is situated within membrane structures through the insertion of palmitoylated residues into the lipid bilayer and its interaction with anionic phospholipids. This enzyme is responsible for catalyzing the hydrolysis of sphingomyelin, leading to the formation of ceramide and phosphocholine.^461^ An augmented visceral WAT mass elevates the susceptibility to cardiovascular diseases,^462^ and visceral adipose tissue plays a crucial role in ceramide synthesis, serving as a primary site for its production.^463^ Ceramide, a key component of sphingolipids, contributes to insulin resistance and hepatic steatosis.^464,465^ Plasma ceramide levels serve as a biomarker for atherosclerosis, promoting inflammation and apoptosis in endothelial cells, vascular smooth muscle cells and macrophages.^466–469^ Ceramide also enhances hepatic lipid synthesis and very low density lipoprotein (VLDL) secretion, leading to dyslipidemia.^470,471^ An inhibitory effect of ceramide on hepatic cholesterol elimination, achieved through the suppression of hepatic *Cyp7a1* and *Abcg5/8*, was observed.^92,460^ C16:0 ceramide administration increased ceramide species in epididymal WAT and plasma and suppressed hepatic CYP7A1 expression via ERK signaling. This impaired hepatic cholesterol elimination, causing dyslipidemia. Different ceramide species dynamically interconvert and mediate adipose-liver interaction in vivo.^92^ Furthermore, cholesterol, a vital component in atherosclerosis, depends on oxygen for its synthesis.^472^ Paradoxically, hypoxia elevates cholesterol levels, heightening the risk of cardiovascular disease.^473^ Hepatic *Cyp7a1* gene expression decreases due to elevated E4BP4, likely through HIF-2α activation and subsequent Rev-erbα inhibition, a circadian-related molecule (Fig. 5c).^14^ Hepatic HIF-2α activation (instead of HIF-1α) cause hypercholesterolemia. In addition, humans also showed an improvement in hypercholesterolemia following mild cold exposure,^474^ while chronic exposure to a thermoneutral environment (30 °C) exacerbates atherosclerosis through the enhancement of vascular inflammation.^475^ However, acute extreme cold (4 °C) may trigger excessive breakdown of fatty tissue (adipose lipolysis) and abnormal movement of monocytes, leading to the development of dyslipidemia, inflammation, and unstable plaques.^476^ Furthermore, an intriguing relationship has been found between cardiovascular disease mortality and ambient temperature, demonstrating an inverse J-shaped association.^106^ This association indicates that moderate cold temperatures confer the lowest risk of cardiovascular disease mortality, while both extreme cold and hot temperatures elevate the risk.

Thermogenic adipocytes convert energy to heat, defending body temperature in cold environments and diverting excess fat. This adaptive thermogenesis significantly contributes to energy expenditure.^477^ Mammals basically have three types of adipocytes based on thermogenic capacity: nonthermogenic white adipocytes, constitutively brown adipocytes, and inducible beige adipocytes.^478^ White adipocytes store excess energy as triglycerides (TG) with large, unilocular lipid droplets that maximize triglyceride storage capacity, whereas brown and beige adipocytes efficiently convert energy into heat with small, multilocular lipid droplets that facilitate β-oxidation.^479^ Besides brown adipose tissue (BAT), the presence of beige adipocytes in WAT has attracted attention as a viable therapeutic target. When stimulated, these beige adipocytes exhibit inducible properties and can dissipate surplus energy as heat.^480^ Under iron-depleted conditions, IRP1 and IRP2 regulate iron homeostasis by overcoming inhibitory effects caused by Fe-S cluster binding and proteasomal degradation, respectively. This promotes IRP/IRE interactions, which was proportionally increased in accordance with the thermogenic function of the adipose depot.^481^ Enhanced IRP/IRE-induced iron influx into mitochondria is vital for thermogenic brown and beige adipocyte differentiation. Iron metabolism in adipose tissue is intricately connected to endocrine functions. In humans, the levels of serum ferritin and transferrin exhibit an inverse correlation with adiponectin and insulin sensitivity.^482,483^ The β_3_-adrenergic receptors (ADRB3) activation induces beige adipogenesis, known as "browning," promoting mitochondrial biogenesis.^484^ Conversely, loss of thermogenic function, or "whitening," reduces mitochondrial content via autophagic degradation.^485^ Adipose iron content can be elevated in response to ADRB3 stimulation-induced adipose tissue browning, but reduced under thermoneutral conditions.^481^ Furthermore, beige adipogenesis is linked to a rapid increase in systemic oxygen consumption^486^ and hypoxia in adipose tissue^487,488^. ADRB3 activation triggers two distinct yet coordinated iron-regulatory pathways. The first pathway establishes an intracellular iron gradient to facilitate the binding of IRP (both IRP1 and IRP2) to IRE in adipocytes. Meanwhile, the second pathway induces acute hypoxia, releasing stored iron into the circulation (Fig. 5c).^426,481^ Adipocytes appear to induce a pseudohypoxic state through ADRB3-mediated adaptive responses involving cytosolic iron deprivation and mitochondrial ROS production. This process leads to HIF-1α stabilization independent of oxygen levels, effectively creating an environment mimicking hypoxia. In the context of this acute hypoxia, the significance of HIF-1α and its induction of TFR1 (importing iron into cells) lies in their crucial role in mitochondrial development within brown and beige adipose tissue, as well as the establishment of thermogenic function.^426,488,489^ In particular, cold treatment in beige adipocytes specifically stabilizes HIF-1α and increases the expression of the *Tfr1* gene. In interscapular BAT, the absence of *Tfr1* leads to the conversion of brown preadipocytes into white adipocytes and muscle cells. However, a prolonged low-iron diet does not replicate this transdifferentiation effect observed in *Tfr1*-deficient mice.^488^ Following the original responses of adipose tissue, the kidneys detect the acute hypoxia in the inguinal WAT induced by ADRB3. This triggers systemic hypoxic responses mediated by HIF-2α, promoting stress erythropoiesis in the spleen.^426^ The accompanied proliferating erythroblast releases erythroferrone (ERFE), which then suppresses hepcidin production in the liver. Liver cells lack ADRB3 and, consequently, cannot react to ADRB3 signaling. As mentioned above, suppressing hepcidin production enhances the influx of intestinal iron into the portal circulation and facilitates the release of iron from storage cells (such as in liver and spleen).^490^ Furthermore, regardless of inguinal WAT hypoxia’s nature following ADRB3 stimulation, it appears that HIF-2α-dependent hypoxic responses in the kidneys are necessary for optimal beige fat development.^426^ In sum, acute hypoxia and stress-induced erythropoiesis aid iron acquisition for white-to-beige fat conversion, during which ADRB3 stimulation activates HIF-2α, EPO production, and splenic erythroid maturation, suppressing hepcidin. Hepcidin down-regulation is vital for beige fat development. Central obesity may hinder beige thermogenesis due to increased hepcidin levels, which inhibit iron mobilization needed for beige conversion.^491^

Collectively, there are two major trends in the outcome of adipose tissue associated with hypoxia-inducible factors in the body. One trend is "solidification," wherein the incapacitation of adipose tissue and its surrounding tissues occurs through the promotion of inflammation and fibrosis. The other trend is "melting," which involves enhancing fat thermogenesis or energy production to redistribute and optimize the utilization of internal resources. Cryogenic therapy, which exposes individuals to extreme cold vapor for a short time, has been found to reduce fat mass by increasing thermogenic energy loss from subcutaneous fat.^492,493^ This cold-induced thermogenic process also leads to reduced hepcidin levels,^494^ indicating a potential link between iron mobilization and adaptive thermogenesis in humans. Considering this, it is worth exploring whether this "fat-burning" approach also holds benefits for the cardiovascular system.

## Therapeutic targets and clinical research progress

**Fig. 6:**
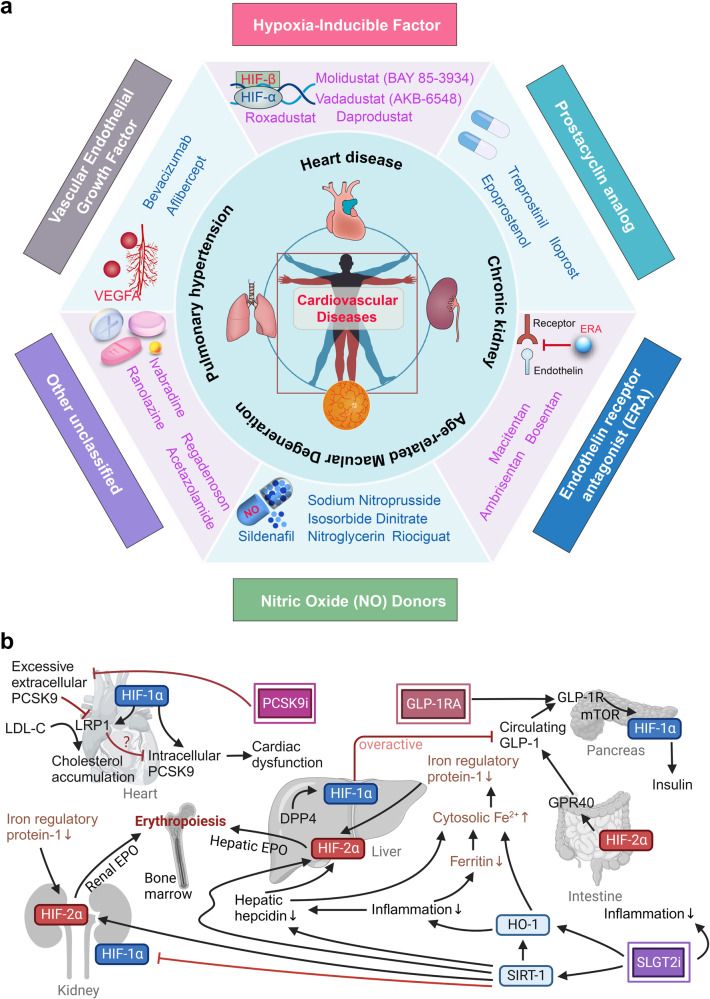
Development of drugs targeting hypoxia signaling in cardiovascular diseases. **a** Pharmacological agents directly or indirectly associated with HIF signaling. **b** HIFs relationship with GLP-1RA, SLGT2i, and PCSK9i. (Created with *BioRender.com*). Abbreviations: DPP4 dipeptidyl peptidase-4, GLP-1 glucagon-like peptide-1, GLP-1R glucagon-like peptide-1 receptor, GLP-1RA glucagon-like peptide-1 receptor agonist, GPR40 G protein-coupled receptor 40, LDL-C low-density lipoprotein cholesterol, LRP1 low-density lipoprotein receptor-related protein 1, PCSK9 proprotein convertase subtilisin/kexin type 9, PCSK9i proprotein convertase subtilisin/kexin type 9 inhibitor, SLGT2i sodium-glucose cotransporter-2 inhibitor

Hypoxia signaling plays a crucial role in various physiological and pathological conditions, such as cancer, cardiovascular diseases, and ischemic injuries.^495,496^ To harness the therapeutic potential of hypoxia signaling, researchers have focused on developing drugs that target specific components of this pathway (Table 2, Fig. 6a). The HIF is a crucial therapeutic target and serves as a master regulator of hypoxia signaling.^496^ In the context of cardiovascular disease, drugs developed based on targeting HIF have demonstrated promising potential. Researchers have extensively explored modulators of HIF-α or coactivators, or related upstream and downstream effector molecules as potential drugs in cardiovascular field.^497,498^ In this review, drugs directly related to HIFs refer to those that directly affect the HIF pathway, including drugs that stabilize HIFs or inhibit the degradation of HIFs.^497,499^ On the other hand, drugs indirectly associated with HIFs are those that target downstream or effector molecules of HIFs, or drugs that ameliorate tissue hypoxia. Apart from treatment methods that directly target the HIF pathway, there is another category of strategies that achieve therapeutic effects by intervening in pathways related to improving the body’s oxygen supply. For instance, treatment methods such as NO donors targeting vasodilation and VEGF promoting angiogenesis can improve angiogenic capacity and tissue perfusion in patients with ischemic cardiovascular diseases to a certain extent.

**Table 2:** Development of hypoxia signaling-related pharmacological agents: potential therapeutic targets and small molecule candidates

Small molecule drugs/candidates	Categorization (therapeutic target)	Description	Disease	First approval
Roxadustat (FG-4592, ASP1517, AZD9941)	Directly (influencing HIF signaling)	HIF-PHI: inhibition (PHD1-3), stablization (HIF-1α, HIF-2α)	Anemia, CKD	2018 in China
Daprodustat (GSK1278863)	Directly (influencing HIF signaling)	HIF-PHI: inhibition (PHD1-3), stablization (HIF-1α, HIF-2α)	Anemia, CKD	2020 in Japan
Vadadustat (MT-6548, AKB-6548)	Directly (influencing HIF signaling)	HIF-PHI: inhibition (PHD3 prefered); stablization (HIF-2α>HIF-1α)	Anemia, CKD	2020 in Japan
Enarodustat (JTZ-951)	Directly (influencing HIF signaling)	HIF-PHI: inhibition (PHD1-3); stablization (HIF-1α, HIF-2α)	Anemia, CKD	2020 in Japan
Molidustat (BAY 85-3934)	Directly (influencing HIF signaling)	HIF-PHI: inhibition (PHD1-3, particularly PHD3); stablization (HIF-2α, HIF-1α)	Anemia, CKD	2021 in Japan
Desidustat (ZYAN1)	Directly (influencing HIF signaling)	HIF-PHI: inhibition (PHD1-3); stablization (HIF-1α, HIF-2α)	Anemia, CKD	2022 in India
R59949	Directly (influencing HIF signaling)	PHD2 activation	PAH	N/A
2-methoxyestradiol	Directly (influencing HIF signaling)	HIF-1α inhibition (at the level of protein synthesis)	PAH	N/A
Topotecan	Directly (influencing HIF signaling)	HIF-1α inhibition (at the level of mRNA)	PAH	N/A
Digoxin	Directly (influencing HIF signaling)	HIF-1α inhibition (at the level of protein synthesis)	PAH	N/A
Anti-CD146 monoclonal antibody, AA98	Directly (influencing HIF signaling)	HIF-1α inhibition (targeting the upstream molecules)	PAH	N/A
Caffeic acid phenethyl ester	Directly (influencing HIF signaling)	HIF-1α inhibition (at the level of protein synthesis)	PAH	N/A
Celastramycin	Directly (influencing HIF signaling)	HIF-1α inhibition (at the level of protein synthesis)	PAH	N/A
YC-1	Directly (influencing HIF signaling)	HIF-1α inhibition (at the level of protein buildup and transcriptional activity)	PAH	N/A
HIF-2α-ASO	Directly (influencing HIF signaling)	HIF-2α inhibition	PAH	N/A
PT2567	Directly (influencing HIF signaling)	HIF-2α inhibition (at the level of heterodimerization and DNA binding)	PAH	N/A
C76	Directly (influencing HIF signaling)	HIF-2α inhibition (at the level of mRNA)	PAH	N/A
Apigenin	Directly (influencing HIF signaling)	HIF-1α inhibition (targeting the upstream molecules)	PAH	N/A
Juglone	Directly (influencing HIF signaling)	HIF-1α inhibition (at the level of protein synthesis)	PAH	N/A
Resveratrol	Directly (influencing HIF signaling)	HIF-1α inhibition (targeting the upstream molecules)	PAH	N/A
Astragaloside IV	Directly (influencing HIF signaling)	HIF-1α inhibition (at the level of protein synthesis)	PAH	N/A
Tagitinin C (1)	Directly (influencing HIF signaling)	HIF-1β inhibition	PAH	N/A
Luteolin	Directly (influencing HIF signaling)	HIF-2α inhibition (at the level of protein synthesis)	PAH	N/A
Bevacizumab	Indirectly (VEGF)	Monoclonal antibody targeting VEGF	Cancer, AMD	N/A
Aflibercept	Indirectly (VEGF)	Fusion protein targeting VEGF	Cancer, AMD	N/A
Tirapazamine/PR-104A	Indirectly (HAPs)	HAP	Cancer	N/A
Regadenoson	Indirectly (adenosine receptors)	Adenosine Receptor Agonizts	cardiac imaging	N/A
Acetazolamide	Indirectly (carbonic Anhydrase)	Carbonic Anhydrase Inhibitor	Altitude Sickness, Edema	N/A
Nitroglycerin	Indirectly (NO donors)	Promote vasodilation, enhance oxygen supply	Angina	1879
Isosorbide Dinitrate	Indirectly (NO donors)	Promote vasodilation, enhance oxygen supply	Angina	1962
Sodium Nitroprusside	Indirectly (NO donors)	Increased cGMP and vasodilation	Hypertensive emergencies	1974
Sildenafil/Tadalafil	Indirectly (PDE5 inhibitors)	Increased cGMP and vasodilation (sildenafil may also inhibiting HIF-2α and HIF-1β)	PAH	1998/2013
Riociguat	Indirectly (sGC stimulator)	Raising NO levels for vasodilation	PAH	2013
Treprostinil/Epoprostenol/Iloprost	Indirectly (prostacyclin analog)	Promote vasodilation, enhance oxygen supply	PAH	2002/1995/2004
Selexipag	Indirectly (prostacyclin receptor agonist)	Promote vasodilation, enhance oxygen supply	PAH	2015
Bosentan/Ambrisentan/Macitentan	Indirectly (endothelin receptor antagonist)	Lowers vasoconstriction and pulmonary resistance	PAH	2001/2007/2013
Ivabradine	Indirectly (HCN channel blocker)	Lowering heart rate and enhancing myocardial oxygen utilization	Chronic stable angina	2015
Ranibizumab	Indirectly (VEGF)	Anti-VEGF monoclonal antibody fragment	Retinal vascular disorders	2006

The retrieval time is up to Sep 2023 *AMD* age-related Macular Degeneration, *ASO* antisense oligonucleotide, *CKD* chronic kidney disease, *cGMP* cyclic guanosine monophosphate, *HAPs* hypoxia-activated prodrugs, *HIF* hypoxia-inducible factor, *HIF-PHIs* HIF-prolyl hydroxylase inhibitors, *NO* nitric oxide, *PAH* pulmonary arterial hypertension, *PDE5* phosphodiesterase-5, *sGC* soluble guanylate cyclase, *VEGF* vascular endothelial growth factor, *N/A* not available

### Drugs directly targeting the core hypoxia signaling

#### PHD modulators in integrated cardiorenal management

Cardiovascular diseases and chronic kidney disease (CKD) share mechanisms and mutually worsen each other. Patients with both heart and kidney conditions not only have significantly higher overall cardiovascular event risk but also a markedly increased risk of cardiovascular and all-cause mortality. Therefore, managing kidney complications alongside cardiovascular disease treatment is equally crucial to slow down the progression of cardiovascular disease and improve long-term patient outcomes. The combined treatment of heart and kidney conditions is of utmost importance in comprehensive panvascular disease management.^500,501^ Directly related to the hypoxia signaling pathway, PHD inhibitors (also known as hypoxia-inducible factor prolyl hydroxylase inhibitor, HIF-PHI), are the earliest and most classic small molecules used in clinical treatment of chronic renal anemia.^27^ Notably, a mature type of drug for inhibiting PHD, “-dustat” (Table 2), promising for the corresponding clinical use in kideny diseases, is now further investigated for its cardiovascular benefits.^502–504^ Molidustat, a promising HIF stabilizer, has been being assessed as a potential therapy for renal anemia in CKDs.^505^ its potential benefits in a rat model of diabetic cardiomyopathy has been demonstrated that molidustat-induced activation of HIF-1α effectively rescues the impaired hypoxic response and restores metabolic dysfunction in the diabetic heart.^497^ In another rat model of MI, administration of the HIF-PHI GSK360A demonstrated a mitigation of MI and improvement in cardiac function.^506^ Moreover, in a mouse model of myocardial I/R, pretreatment with roxadustat (the first small-molecule HIF-PHI) significantly has been observed to decrease the size of cardiac infarction,^507^ indicating its potential therapeutic efficacy for cardiac I/R injury.

Furthermore, theoretically, improving anemia may have the potential to enhance cardiac function in patients with heart failure and concurrent anemia. However, darbepoetin alfa, a synthetic analog of human EPO, increases RBC production and relieves hypoxia in renal anemia treatment, but a clinical trial (NCT00358215) investigating its use for improving cardiac function in renal anemia yielded disappointing results.^508,509^ While some research suggests that this may result from excessive activation of darbepoetin alfa, pushing EPO levels far beyond physiological limits, HIF-PHI is thought to moderately activate EPO without disrupting endogenous homeostasis, achieving therapeutic effects.^6,27^ Nevertheless, there are still incongruous outcomes observed. Some phase 3 clinical trials (NCT02648347 and NCT02680574) evaluating the efficacy of vadadustat in non-dialysis-dependent CKD patients, and it was observed that the group receiving vadadustat treatment experienced a higher occurrence of the composite endpoint comprising death, myocardial infarction, and stroke.^510^ Therefore, further comprehensive research, both at the fundamental and clinical levels, is required to determine whether the next generation of HIF-PHIs can effectively improve the prognosis of heart failure in the context of CKD and anemia.

Currently, the focus of PHD inhibitor research is on enhancing their efficiency in boosting EPO or achieving better penetration into specific sites, such as crossing the blood-brain barrier. For instance, IOX-2 was developed with a high affinity (IC50 = 22.0 nM) for increasing endogenous EPO levels,^511^ and IOX-4 induced HIF-α in various human cells (HeLa, Hep3B, and RCC4) as well as in the brain tissue of wild-type mice.^512^ This stabilization of HIF in brain tissue is particularly promising for addressing cerebral conditions like stroke. Various techniques such as affinity selection-mass spectrometry (AS-MS), a click-chemistry-based approach, and high-throughput screening (HTS) methods are currently employed in the development of improved PHD inhibitors with enhanced oral absorption, faster onset of action, superior pharmacokinetics, and enhanced safety profiles. Using AS-MS screening, potent PHD inhibitors, a series of triazaspiro[4.5]-decanones were discovered with short-acting PHD inhibition and improved in vivo efficacy.^513^ Through click chemistry, [3-Hydroxy-5-(1*H*-1,2,3-triazol-4-yl)picolinoyl]glycines, an oral inhibitor was developed with almost 10 times (IC50 of 62.3 nM) more potent than roxadustat in vitro, promising a safe anemia treatment.^514^ Furthermore, the HTS method identified a new group of promising PHD pan inhibitors, exemplified by MK-8617.^515^ Unlike previously mentioned inhibitors, MK-8617 lacks a carbonyl acid group, and molecular modeling reveals that its heterobiaryl moieties chelate Fe^2+^ and one methoxy-substituted aryl ring interacts with Arg322 through cation-π interactions in PHD2. MK-8617 exhibits enhanced potency and bioavailability in rats, with a monkey half-life of 10-19 h, making it a favorable candidate for in vivo PHD targeting.^515^ Overall, current small-molecule PHD inhibitors mainly fall into two categories: i) noncompetitive and nonselective iron chelators and ii) competitive and selective 2-OG mimetics. Iron chelators lack selectivity as they can interact with all iron-containing proteins, potentially leading to harmful side effects like heart failure, liver cirrhosis, and fibrosis. In dialysis-dependent CKD anemia patients, there were no significant differences found in this network meta-analysis between HIF-PHIs and erythropoiesis-stimulating agents (ESAs) in terms of various risks (including cardiovascular events, hyperkalemia, cancer, hypotension, muscle spasms, and etc.).^516^ However, for long-term HIF-PHI use, personalized and selective approaches are crucial, particularly for cancer patients with chemotherapy-related anemia.^6^ Thus, competitive and selective PHD inhibitors are still being eagerly wanted.

Furthermore, blocking VHL and HIF-α interaction can enhance HIF-α and target genes, a viable approach for CKD anemia treatment by enhancing endogenous EPO.^517,518^ Compared to PHD inhibitors, VHL inhibitors may effectively prevent off-target effects unrelated to HIF signaling. The HyP-564 residue on HIF-1α plays a crucial role in its interaction with VHL.^519^ Both molecular growth and docking strategies have been employed to facilitate the selection of optimal hydroxyproline analogs.^520^ VH298, a potent VHL inhibitor, effectively induces HIF-α hydroxylation under hypoxic conditions in HeLa, U2OS, and CTL cell lines.^521^ A previous study found that VH298-loaded extracellular vesicles improve diabetic wound healing by enhancing angiogenesis through the HIF-1α/VEGFA signaling pathway.^522^ Furthermore, a highly isoform selective manner without inducing a hypoxic response could be achieved by Homo-proteolysis-targeting chimeras (PROTACs). In the context of PROTACs, the idea is to create molecules that can bind to a target protein of interest and an E3 ubiquitin ligase simultaneously. This dual binding triggers the ubiquitination of the target protein by the E3 ligase, marking it for degradation within the cell’s proteasome. Homo-PROTACs, like PROTACs, degrade proteins. The difference is that in Homo-PROTACs, two identical small molecules bind to both target proteins, acting as a bridge that recruits an enzyme for protein degradation. Considering the crystal structure of VH298 in complex with VHL: ElonginB: ElonginC (VBC) and cellular permeability, a VHL Homo-PROTAC CM11 has been developed with high avidity to dimerize VHL and could trigger prominent, efficient, and self-degradation of VHL mediated by proteasomes in vitro.^523,524^ These findings expand our understanding of the currently available PROTAC-type scaffolds that could be beneficial for targeted degradation of various other therapeutically proteins.

#### HIF modulation in cardiovascular management

Balancing moderate HIF-1α activity while promoting HIF-2α for stable angiogenesis and increased EPO could be a promising direction in cardiovascular diseases or overnutrition diseases.^6,15,139^ This concept is exemplified in heart and kidney management, heart failure treatment, and novel lipid-lowering drugs (Fig. 6b). Type 2 diabetes mellitus and CKD are independent cardiovascular risk factors, central to screening and managing cardiovascular disease. At the ESC Congress 2023, comprehensive management of patients with diabetes comorbid with cardiovascular disease and CKD garnered significant attention, emphasizing the treatment principle of "aiming to improve heart and kidney outcomes."^500^ Glucagon-like peptide-1 receptor agonist (GLP-1RA) has been proposed as Class I recommendation to reduce cardiovascular risk independent of glucose control. Glucagon-like peptide-1 (GLP-1) is released from intestinal L-cells in response to nutrients like monosaccharides and lipids through unique mechanisms.^525,526^ The steep oxygen gradient in the intestines is vital for maintaining intestinal health, and its disruption can lead to barrier dysfunction, pathogen colonization, and inflammatory diseases.^527^ Activation of intestinal HIF-2α signaling boosts GLP-1 levels via the HIF-2α–G-protein–coupled receptor (GPR)40 axis and reduces diet-induced metabolic issues like visceral fat, glucose problems, and liver fat buildup.^526^ Intriguingly, vertical sleeve gastrectomy (VSG), a highly effective and long-lasting solution for obesity and type 2 diabetes, boosts gut HIF-2α signaling, which reduces weight, enhances glucose control, and boosts GLP-1 secretion.^528^ Active circulating GLP-1 levels may be low in obese patients. Obesity raises circulating adipokines, like the leptin/adiponectin ratio and free fatty acids, activating liver HIF-1α expression. This increases liver dipeptidyl peptidase-4 (DPP4) and sinusoidal flow resistance, reducing active GLP-1. Removing HIF-2α from liver cells doesn’t affect DPP4 but improves hepatic steatosis.^529^ Meanwhile, repressing hepatic DPP-4 with saxagliptin can also reduce HIF-1α, mitigate fatty liver and improve glucose tolerance.^530^ Furthermore, reducing overactive HIF-1α in obese adipose tissue improves glucose tolerance by enhancing insulin secretion through the GLP-1 pathway and reducing inflammation and macrophage infiltration.^531^ In addition, GLP-1 boosts gene expression in pancreatic β-cells through two phases: one immediately through cyclic AMP Response Element-Binding protein (CREB) and another later through HIF, where HIF could be activated by GLP-1 via the IRS2-AKT pathway and mTOR kinase.^532^ GLP-1 then boosts insulin secretion by pancreatic β-cells through glucose-dependent HIF-1α-driven glycolysis.^533,534^ Moreover, metabolic stress triggers HIF-2α in β-cells, promoting antioxidant gene activation (like *Sod2* and *Cat*) to shield against mitochondrial ROS and prevent damage.^535^ This suggests HIF plays a role in GLP-1’s long-term benefits for pancreatic islet function by enhancing cell defense and metabolic programs that support β-cells viability. Furthermore, GLP-1 appears to promote elastogenesis, hinder the fibrotic process and thus contrbute to cardiac recovery following acute MI or chronic heart failure.^536^ Recently in the STEP-HFpEF trial (NCT04788511), a peptidic GLP-1RA semaglutide (2.4 mg) outperformed a placebo in treating obese heart failure patients with preserved ejection fraction with more symptom reduction, exercise function improvement, and weight loss.^537^

Moreover, the sodium/glucose cotransporter 2 inhibitor (SGLT2i) is now the only medication that covers the full range of heart failure care, thanks to its potential for cardiac regeneration.^500,538^ High glucose, advanced glycation end products (AGEs), mTOR, and hypoxia overactivate HIF-1α, while SIRT1 suppression downregulates HIF-2α. in diabetes.^15^ And SGLT2is mimic fasting, activating cellular nutrient deprivation signals, dampening stress and inflammation pathways, underlying the stimulation of sirtuin-1 (SIRT-1) and heme oxygenase-1.^539^ Then, reduced inflammation-related hepcidin and ferritin expression elevates reactive ferrous iron (Fe^2+^), promoting SIRT1’s activation on HIF-2α in the liver and kidney, while while reducing HIF-1α in the kidney. This triggers moderate hepatic and renal EPO production and corresponding changes in hemoglobin levels indicate SGLT2is’ efficacy in lowering the risk of cardiovascular death and heart failure hospitalizations.^139,540,541^ Also, using the HIF-2α inhibitor PT2399 countered the effects of dapagliflozin on cardiac fibrosis and inflammation in arrhythmogenic cardiomyopathy.^542^ In addition, proprotein convertase subtilisin/kexin type 9 inhibitors (PCSK9is) are new drugs for high cholesterol and heart problems. Acute MI boosts PCSK9 expression in cardiomyocytes through elevated HIF-1α levels. In cardiomyocytes, HIF-1α siRNA reduced myocardial PCSK9 expression and autophagy signaling under hypoxia, and two PCSK9is, Pep2-8 and EGF-A, improved heart function and reduced infarct size in mice with left anterior descending coronary artery ligation.^543^ Furthermore, LRP1 (also known as CD91) is constitutively expressed in all heart-resident cell types and its expression significantly increases during hypoxemia and/or ischemia through HIF-1α activation.^544,545^ Adult rat cardiomyocytes treated with recombinant PCSK9, not endogenous PCSK9 knockdown by siRNA, led to reduced LRP1 protein levels. This disruption subsequently affected LRP1-C1q/TNF-related protein (CTRP) mediated glucose metabolism and mitochondrial biogenesis.^546^ However, when activated by low-density lipoprotein cholesterol (LDL-C), these receptors LRP1s in cardiomyocytes seem to be responsible for cholesterol accumulation.^545^

While these popular drugs are known to influence HIF-related pathways, the intricacies of HIF pathway regulation make it possible for some drugs to inadvertently impact other pathways, causing off-target effects. Consequently, there is still a significant research focus on developing drug molecules that specifically target HIF-related structures. One strategy to block HIF-1 involves disrupting the complex of HIF-1α C-TAD and the cysteine/histidine-rich region CH1 domain of CBP/p300 (also known as the Eck’s group) using α-helix mimetics.^547,548^ Chetomin derivatives and KCN directly inhibit the Eck’s group without affecting intracellular HIF-1α levels. Advanced chetomin analogs like ETP2 and ETP3 show reduced toxicity to normal cells compared to natural chetomin.^549,550^ Another inhibitor of the Eck’s group, arylsulfonamide KCN1, was identified in vitro through a surface plasmon resonance (SPR) assay.^551^ In vitro, KCN1 hampers pancreatic cancer cell growth and triggers cell cycle arrest in a dose-dependent manner, with good pharmacological properties.^552,553^ Chetomin derivatives and KCNs are able to minimize in vivo toxicity, suggesting their potential for treating conditions with HIF overexpression, like vascular diseases and cancers. Furthermore, researchers have crafted peptoid inhibitors to selectively target the binding of HIF-1α with p300 by mimicking energetically important protein residues, such as Leu818, Leu822, Asp823, and Gln824.^554^ Through structure-activity relationship (SAR) analysis, a designed peptidomimetic HBS1 effectively inhibited tumor growth in an renal cell carcinoma (RCC) xenograft mouse model.^554^ Computational and structural analyses have identified the CH1 domain of p300/CBP interacting with HIF-1α helix residues. As a result, peptoid OHM1, mimicking essential amino acids Leu818, Leu822, and Gln824, demonstrates potent and selective inhibition of the Erk’s group in both in vitro and in vivo settings.^555^

Furthermore, the precise targeting of specific HIF-α subunits using small molecules is a promising avenue of research, considering the distinct functions of HIF-1α and HIF-2α. However, it’s worth noting that HIF lacks a known ligand-binding domain, making the development of subtype-selective small-molecule inhibitors challenging due to the high similarity between HIF-1α and HIF-2α.^556^ Intriguingly, the HIF-2α PAS-B domain harbors a substantial concealed cavity (290 Å^3^) within its hydrophobic core, which is occupied by water molecules.^557^ The cavity was first detected by Gardner et al.^52^ in 2009 after screening 200,000 structurally diverse small molecules by NMR-based ligand binding assay. Thereafter, Met252^558^ and His293^559^ of the cavity were found crucial for allosteric inhibition and hydrogen bond formation respectively. Antagonists to the HIF-2α-PAS-B cavity allosterically disrupt its heterodimerization with HIF-1β, which is further delineated by Wu et al.^560^ in 2019. PT2385 and MK-6482 are the most active and representative inhibitors of HIF-2α/HIF-1β dimerization for the treatment of RCC.^561–563^ Typically, PT2385’s hydroxyl group forms hydrogen bonds with Tyr281, a water molecule, and His293. This shifts three HIF-2α residues (Met252, His293, Tyr278) but leads to a clash between Tyr278 and Phe446 in HIF-1β, disrupting the HIF-2α: HIF-1β dimer and inhibiting HIF-2α transcription.^564^ No adverse effect on the cardiovascular system has been so far reported on PT2385.^565^

Collectively, balancing HIF-1α and promoting HIF-2α activity offers promising prospects in addressing cardiovascular diseases and conditions like diabetes and chronic kidney disease. Drugs like GLP-1RA, SGLT2i, and PCSK9i are showing potential in modulating HIF-related pathways for improved cardiovascular outcomes. Moreover, the development of small molecule inhibitors targeting specific HIF-α subunits, such as PT2385 for HIF-2α, holds therapeutic promise. Additionally, given the previously mentioned unique sensitivity of the lungs to hypoxia and the specific role of HIF-2α in initiating the pathological progression of PAH, numerous small molecule candidates targeting PHD or HIF have been experimentally demonstrated to alleviate PAH (Table 2).^27,128,566–570^ The concurrent utilization of a tumor suppressor p53 agonist and an HIF-2α antagonist has the potential to specifically and concurrently hinder the hypoxia-triggered proliferation of pulmonary artery smooth muscle cells and the apoptosis of pulmonary artery endothelial cells. This approach aims to address the adverse effects associated with single-agent therapies for PAH.^571^ However, careful consideration of potential off-target effects and the intricate regulation of HIF pathways is essential in drug development.

### Drugs indirectly targeting the core hypoxia signaling

In the development of hypoxia-related drugs, targeting specific physicochemical structures for precise modulation of the HIF signaling pathways is crucial. However, sometimes, directly controlling effector molecules upstream or downstream of the HIF signaling pathway can be more effective. Or rather, "directly related to HIF signaling" refers to drugs or small molecular compounds developed to influence HIFs signaling. Conversely, "indirectly related to HIF signaling " pertains to drugs capable of ameliorating the hypoxic state in cardiovascular diseases, such as nitric oxide, PDE5, sGC, and others. These drugs primarily enhance oxygen exchange within the circulatory system to address hypoxia in heart tissues.

#### VEGF-related candidates

Alternative revascularization strategies hold great promise in the management of ischemic diseases, specifically ischemic heart disease, by emphasizing the stimulation of neovascularization to ensure vital oxygen and nutrient delivery during tissue ischemia and repair.^572^ Hypoxia plays a pivotal role as a major driver of angiogenesis, triggering the synthesis of diverse angiogenic factors, including VEGF, which acts as a hypoxia-responsive mitogen and modulator of vascular permeability.^573,574^ Maintaining the activation of the HIF signaling pathway within a normal range has been demonstrated to mildly enhance the expression of EPO and VEGF. This results in improved metabolic and cardiovascular health, including better glucose tolerance, decreased total cholesterol and blood pressure, reduced toxic metabolites, and a lower inflammatory burden. Additionally, it prevents microvascular rarefaction and promotes rejuvenation of vascular health, contributing to overall well-being.^439,440^ Currently, researchers are actively developing drugs to target VEGF for treating ischemic heart disease, with a focus on boosting VEGF levels in affected areas. Animal studies have revealed that the utilization of calcium alginate microsphere patches for delivering VEGF to rat hearts following myocardial injury promotes local angiogenesis within the infarcted heart, leading to improved cardiac function.^575^ Similarly, another investigation employing targeted nanoparticles for VEGF delivery to myocardial tissue has demonstrated comparable reparative effects.^576^ Additionally, promising outcomes have been observed in clinical trials. A registered study demonstrated that high-dose plasmid-mediated VEGF gene expression in ischemic myocardial tissue can enhance the prognosis of ischemic heart disease.^577^ Collectively, these findings underscore the remarkable therapeutic potential of VEGF in cardiovascular disorders, particularly in the context of ischemic heart disease. Unlike ischemic diseases, the abundant blood supply in malignant tumors and retinal/choroidal vascular diseases presents a unique challenge to disease treatment.^578–580^ Currently, several drugs targeting VEGF or VEGF receptors (VEGFR) have been approved for inhibiting angiogenesis in conditions such as tumors.^581^ The former includes bevacizumab, a humanized monoclonal antibody that neutralizes VEGF-A, while the latter comprises small molecule kinase inhibitors such as sorafenib, sunitinib, pazopanib, vandetanib, axitinib, regorafenib, lenvatinib, nintedanib, and cabozantinib, which specifically inhibit VEGFR.^582^ However, it is essential to acknowledge the potential cardiovascular toxicity associated with therapies targeting the VEGF/VEGFR pathway. Multiple studies have reported an elevated risk of hypertension, congestive heart failure, acute coronary syndrome, and MI among patients treated with anti-VEGF monoclonal antibodies like bevacizumab.^583–585^ Hence, balancing maligant angiogenesis and ischemic risks in future VEGF/VEGFR pathway treatments is crucial. Developing precise drugs that target specific VEGF/VEGFR subtypes, like controlled HIF-VEGF pathway activation, may reduce side effects.

#### NO donors

NO donors play a crucial role in the hypoxia pathway.^586–589^ In the presence of low oxygen levels (hypoxia), NO donors have the ability to modulate the hypoxic pathway, facilitating cellular adaptation and mitigating the adverse effects of hypoxia.^588,590^ Consequently, the utilization of NO donors as a therapeutic approach has been implemented in the field of cardiovascular medicine to enhance cardiac blood flow and alleviate symptoms associated with angina. Medications based on NO donors can be classified into different categories. The first category is comprised of nitrate drugs such as nitroglycerin,^591^ isosorbide dinitrate, and isosorbide mononitrate.^592,593^ These drugs undergo metabolism to produce NO, which results in vasodilation, improved blood circulation, and reduced blood pressure. Nitrate drugs are commonly employed in the treatment of ischemic cardiovascular diseases, including angina and acute MI. Another category includes nitroprusside, a type of nitrate drug, for instance, sodium nitroprusside, which also acts as an NO donor. These medications rapidly release NO, leading to vasodilation and blood pressure reduction. Nitroprusside drugs are frequently utilized for urgent blood pressure reduction in severe hypertensive emergencies.^594^ During hypoxia, the levels of NO in the body decrease, which can lead to impaired vasodilation and blood flow regulation. Phosphodiesterase 5 (PDE5) inhibitors work by inhibiting the enzyme PDE5, which breaks down cyclic guanosine monophosphate (cGMP), a signaling molecule involved in vasodilation.^595^ By inhibiting PDE5, these medications increase the concentration of cGMP, promoting vasodilation and enhancing blood flow. In cardiovascular diseases characterized by oxygen deprivation, such as ischemic heart disease and pulmonary arterial hypertension, PDE5 inhibitors have been shown to have beneficial effects. They can improve blood flow to the heart and reduce pulmonary artery pressure, thus alleviating symptoms and improving exercise tolerance in patients.^596,597^ Additionally, PDE5 inhibitors can potentially protect the heart by reducing oxygen consumption, preventing platelet aggregation, and moderating inflammation, leading to better cardiac function and fewer adverse cardiovascular events.^598^

Prostacyclin analogs, which mimic the naturally occurring prostacyclin, exhibit pharmacological properties including vasodilation, inhibition of platelet aggregation, reduction of vasoconstrictor release, and attenuation of inflammatory responses.^599,600^ Prostacyclin analogs, including iloprost, epoprostenol, treprostinil, and beraprost, have extensive utilization in the management of cardiovascular disorders. These medications manifest therapeutic effects via diverse pharmacological mechanisms. Primarily, acting as agonizts of the prostacyclin receptor, they induce vasodilation, particularly within pulmonary vessels, leading to decreased pulmonary arterial pressure, enhanced blood circulation, and improved oxygen delivery. Secondly, they inhibit platelet activation and aggregation, thus diminishing the likelihood of thrombus formation and preserving vascular patency. Furthermore, Prostacyclin analogs exhibit anti-inflammatory properties by suppressing the generation of inflammatory mediators and mitigating tissue inflammation, consequently contributing to the amelioration of hypoxic conditions.^601^ Substantiated by clinical research and practice, these drugs have demonstrated significant enhancements in symptoms, exercise capacity, and quality of life among patients with cardiovascular diseases.

#### Endothelin receptor antagonists

In pathological conditions, the excessive synthesis of endothelin (ET) and its interaction with specific receptors (endothelin type A receptor and endothelin type B receptor) lead to vasoconstriction, inflammatory responses, and tissue fibrosis, thereby worsening ischemia and hypoxia in organs and tissues.^602^ Both HIF-1 and HIF-2 participate in mediating ET-1 in endothelial and smooth muscle cells.^27^ ET receptor antagonists, through their binding to these receptors and subsequent blockade, effectively suppress ET activity, resulting in the mitigation of vasoconstriction and the amelioration of tissue ischemia and hypoxia. Consequently, these antagonists demonstrate therapeutic efficacy in the treatment of cardiovascular diseases. ET receptor antagonists have extensive applications in various cardiovascular diseases. Firstly, in patients with PAH such as bosentan^603^ and macitentan^604^, ET receptor antagonists exert their effects by reducing pulmonary vascular constriction and permeability, thus leading to a decrease in pulmonary arterial pressure. This therapeutic approach not only improves exercise tolerance, and quality of life, but also delays disease progression. Secondly, these drugs have demonstrated significant efficacy in the treatment of hypertension. For instance, darusentan^605^ and aprocitentan^606^, as ET receptor antagonists, effectively lower blood pressure levels by blocking ET receptors and reducing vascular constriction. Additionally, ET receptor antagonists exhibit potential in treating other cardiovascular diseases, including heart failure.^607^ The excessive activation of ET-1 in the cardiovascular system plays a pivotal role in the development of heart failure. By inhibiting the adverse effects of ET-1 on the heart and blood vessels, these medications have the potential to alleviate heart failure symptoms and improve cardiac function.^608^ Furthermore, ongoing studies are investigating the applications of ET receptor antagonists in conditions such as coronary artery disease, renal diseases, and vascular remodeling. With further advancements in research, the future prospects of ET receptor antagonists in the treatment of cardiovascular diseases are expected to expand significantly.

#### Others

In addition, several other drugs have demonstrated potential in improving tissue and organ hypoxia, and have been widely utilized in cardiac diseases, particularly ischemic heart disease. For instance, ivabradine exerts selective inhibition of the "funny" current (I_f current) in cardiac cells, resulting in reduced heart rate and improved myocardial oxygenation insufficiency.^609,610^ Furthermore, the selective adenosine A_2A_ receptors agonist, regadenoson, exhibits coronary artery dilation and enhances cardiac blood flow, effectively ameliorating cardiac hypoxia.^611^ The adenosine-related pathway is considered the ultimate effector pathway of HIF-dependent cardioprotection.^6^ Ranolazine acts through the inhibition of sodium ion channels in myocardial cells, leading to the attenuation of abnormal sodium/calcium exchange, excessive myocardial contraction, and oxygen consumption.^612^ As a consequence, ranolazine finds application in the treatment of stable angina and other ischemia-related conditions.^613^ Additionally, ongoing animal studies have provided evidence indicating that acetazolamide, a carbonic anhydrase inhibitor, promotes coronary artery dilation and augments myocardial blood supply, thereby improving cardiac hypoxia.

What’s more, natural compounds play critical roles as ~40% of all medicines are natural compounds or semisynthetic derivatives.^614^ The HIF-1 signaling pathways can be inhibited by a plenty of natural compounds. However, the majority of these results were performed in vitro. Among the 22 potential compounds reviewed by Wang et al.,^615^ in vivo anti-cancer effects have been confirmed for only eight of these (acriflavine, andrographolide, bavachinin, celastrol, dihydrotanshinone i, diallyl trisulfide, gliotoxin, isoliquiritigenin). Surprisingly, from these eight compounds, only andrographolide is under clinical investigation.^616^ The potential hurdles encountered in clinical translation include a lack of inhibitory potency, low specificity, poor pharmacokinetic property, toxicity issues, and flaws in clinical trial designs. Nevertheless, many scientists are actively involved in the synthesis of a series of novel compounds based on SARs to overcome these limitations.

Collectively, through a comprehensive understanding and modulation of hypoxia signaling, researchers aim to restore cellular responses to hypoxia and mitigate the deleterious consequences of oxygen deprivation (Table 2, Fig. 6). Ongoing research and trials are essential to confirm drug effectiveness for hypoxia-related disorders. Recent cardiovascular research explores various approaches: small molecules like HIF-PHIs, gene therapies, and cell-based treatments. HIF-PHIs stabilize HIF and are tested in trials for conditions like CKD-related anemia and heart failure.^497^ Gene therapies aim to enhance natural hypoxia responses and are studied for critical limb ischemia and angina, involving the delivery of genes encoding HIF or other molecules involved in hypoxia response to target tissues.^617^ Cell therapies (such as using stem cells or progenitor cells), boosting angiogenesis and tissue repair, are investigated for peripheral artery disease and myocardial infarction.^618^ Research targeting hypoxia signaling for cardiovascular diseases advances through diverse strategies harnessing cellular responses to low oxygen.

## Conclusion and perspective

Collectively, a comprehensive understanding of HIFs in the cardiovascular necessitates a multifaceted approach that encompasses multiple dimensions, spanning time and space, macroscopic and microscopic perspectives, as well as historical, present, and future aspects. And this multi-level regulatory framework for hypoxia signals and mechanisms is called for in the development of therapeutic targets for cardiovascular disease and in clinical practice. Overall, the following three revelations can be drawn.

**i) Balancing HIFs throughout the entire process of hypoxia response, natural selection, and circadian rhythms**. HIFs are central in cardiovascular diseases, governing a dynamic "HIFs switch" sensitive to oxygen levels. HIF-1α and HIF-2α, similar in structure but different in function, take turns in controlling energy metabolism and gene expression in response to hypoxia. In acute hypoxia, HIF-1α takes the lead, driving glycolysis and angiogenesis. In contrast, chronic hypoxia sees HIF-2α stepping forward, promoting the maturation of new blood vessels. Imbalances between HIF-1α and HIF-2α can lead to oxidative stress, inflammation, and fibrosis, contributing to chronic conditions. Therapeutic strategies aim to activate HIF-αs effectively during acute phases and prioritize HIF-2α during chronic phases to restore balance and mitigate disease progression. Moreover, the broader perspective of the "HIFs switch" involves long-term adaptations in high-altitude environments. Populations like Tibetans and Andeans have undergone natural selection, resulting in genetic changes (e.g., *EPAS1*, *EGLN1*, and *PPARA*) that optimize oxygen transport, metabolism, and vascular regulation at high altitudes. High-altitude living involves moderate hypoxia, where the main response factor to changing oxygen levels is HIF-2, linked to EPO production. Populations at high altitudes exhibit varying hemoglobin levels, but overall, adaptive changes in oxygen transport and tissue oxygen use occur due to the HIFs switch dominant by natural selection. Clotting-related factors also change in this context, warranting further research. These adaptations offer insights into human evolution and potential treatments for altitude-related health issues, showcasing the intricate relationship between genetics, hypoxia, and cardiovascular health.

Furthermore, the role of rhythm in the hypoxia pathway cannot be ignored. Current research primarily emphasizes the impact of some natural rhythm alterations on changes in HIF expression levels within the body. The accumulation of circadian and seasonal rhythms (such as hibernation) can shape the breeding patterns and rise-and-fall history of a species. The HIFs-induced metabolic shift to FAO plays a vital role in maintaining basic metabolism and eliminating excessive harmful substances like ROS. A deeper exploration of this mechanism can contribute to the development of "human hibernation technology," which will aid interstellar travel and the exploration of the universe.

**ii) Focus on the epigenetic regulation acquired postnatally at the microscale and the interactions between organs/systems at the macroscale for HIFs regulation**. Hypoxia exerts its influence on the occurrence and progression of cardiovascular diseases through a myriad of epigenetic modifications, including post-translational modifications of histones and non-histone proteins, DNA methylation, and non-coding RNA regulation. PTMs of histones encompass various facets of cardiovascular diseases, including the response of ECs to hypoxic signals in atherosclerosis and PAH. Recently, lactylation, an emerging modification, has commanded attention for its critical role in governing gene transcriptional regulation. Intriguingly, in the context of MI, lactylation stands implicated in myocardial fibrosis and the impairment of cardiac function. Furthermore, lactylation-mediated macrophage polarization assumes a pivotal role in the realm of inflammatory diseases. In the case of PAH, hypoxia-induced glycolytic shift and lactylation modification may facilitate HPVR. Furthermore, the demethylation of the BMPR2 promoter region significantly ameliorates pulmonary vascular and right ventricular remodeling, decreasing RVSP and mPAP. The intratracheal delivery of adeno-associated virus-encapsulated “beneficial genes” represents a promising therapeutic approach. Significantly, an intimate relationship exists between cardiovascular diseases and the intricate landscape of DNA methylation, alongside the post-transcriptional regulatory machinations orchestrated by non-coding RNAs. Also, understanding hypoxia’s impact on cardiovascular diseases requires considering both systemic and local (tissue and organ) effects. Two main pathways drive hypoxia-induced organ/tissue interaction: neurohumoral (systemic secretion) and local stimulation. Cardiovascular processes exhibit circadian influences, affecting cardiovascular events, blood pressure, and myocardial infarction occurrence. The central circadian clock influences cardiovascular health through hormonal factors like adenosine and melatonin. The circadian clock affects drug metabolism, suggesting optimal times for drug administration. Furthermore, the lactate shuttle theory explains the movement of lactate between cells, playing a vital role in energy balance and metabolic efficiency. HIFs create an acidic environment, promoting lactate diffusion. Lactate has complex effects on endothelial cells, affecting angiogenesis and fibrosis in cardiovascular diseases. Timely intervention can shift monocyte responses and regulate histone lactylation for cardiac repair. MCT1 expression influences lactate uptake, favoring self-repair and angiogenesis in the heart. Moreover, understanding the intricate interplay of hypoxia, iron, adipose tissue, and temperature regulation provides insights into cardiovascular health. Balancing these factors may enhance metabolic and cardiovascular traits, offering potential avenues for therapeutic interventions. The interactions between EPO and cardiovascular systems reveal a complex relationship between oxygen regulation, RBC production, and cardiovascular health. EPO, traditionally linked to HIF-1, now also involves HIF-2 in its synthesis. HIF-2 regulates EPO synthesis in the kidneys and liver, affecting serum levels and bone marrow erythropoiesis. This intricate process relies on specific regulatory elements like LIE and KIE. Iron regulation plays a role, impacting HIF-2α expression. These interactions underscore the critical role of oxygen and iron in cardiovascular health. In addition, adipose tissue interactions with cardiovascular diseases reveal the dual nature of obesity. Healthy adipose tissue expansion is protective, while pathological expansion leads to inflammation and fibrosis. Hypoxia-induced HIF-1α worsens adipose tissue dysfunction, exacerbating obesity-related health issues. Conversely, HIF-2α in adipocytes can protect against insulin resistance. The metabolic effects of adipose tissue extend throughout the body, influencing cardiovascular health and cholesterol levels. Moreover, the role of temperature in cardiovascular health is intriguing. Cold exposure can improve adipose tissue function, reducing obesity-related risks. However, extreme cold may have adverse effects, emphasizing the importance of maintaining a balanced environment.

**iii) Balancing HIF-1α activity and promoting HIF-2α activity in drug development**. The current focus on the development of hypoxia-related drugs in the field of cardiovascular diseases primarily revolves around medications that regulate HIF-α or its co-factors and downstream effector molecules within the HIF pathway. Efforts to enhance the effectiveness of PHD inhibitors primarily involve two aspects: a) increasing endogenous EPO production or achieving better penetration into specific sites, such as crossing the blood-brain barrier, and b) utilizing various techniques such as AS-MS, click chemistry-based approaches, and HTS methods to develop improved PHD inhibitors with enhanced oral absorption, faster onset of action, improved pharmacokinetics, and enhanced safety profiles. Currently, small-molecule PHD inhibitors fall into two main categories: non-competitive and non-selective iron chelators and competitive and selective 2-OG mimetics. Iron chelators lack selectivity as they can interact with all iron-containing proteins, potentially leading to harmful side effects like heart failure, liver cirrhosis, and fibrosis. Therefore, there remains a pressing need for selective competitive PHD inhibitors. Furthermore, balancing appropriate activity of HIF-1α while promoting HIF-2α activity holds promise in improving conditions such as cardiovascular diseases and diabetes. Correspondingly, drugs like GLP-1RA, SGLT2i, and PCSK9i have demonstrated potential in modulating pathways related to HIF-1/2α for therapeutic benefits. Also, the development of small molecule inhibitors targeting specific HIF-α subunits, such as PT2385 for HIF-2α, shows therapeutic potential. However, it is crucial to carefully consider potential off-target effects under the complex regulation of the HIF pathway, and potential issues related to drug resistance in the drug development process. Additionally, there is another category of treatment methods that achieve therapeutic effects by intervening in pathways related to improving the body’s oxygen supply. For instance, NO donors and VEGF, which promote vasodilation and angiogenesis, respectively, hold promise in enhancing angiogenic capacity and tissue perfusion in patients with ischemic cardiovascular diseases.

Overall, in the journey of HIF-related targeted therapy from bench side to bedside, healthcare professionals and researchers must acquire a comprehensive understanding of HIF-related mechanisms, the historical development of drug research, and relevant treatment modalities as a foundation for future innovations. In clinical practice, meticulous research into existing pain points and problem identification is essential. This involves tightly integrating clinical medicine with basic research, engineering applications, and information science, while emphasizing both macroscopic and microscopic perspectives and focusing on the spatiotemporal specificity and holistic nature of cardiovascular diseases. Therefore, significant progress in translating and implementing HIF-related treatments, guided by the principle of "from doctors, by engineers/researchers, for patients," can be achieved in the field of cardiovascular medicine.^18^
